# Investigating Anthropogenic and Social Influences on Diet of Semi‐Urban Vervet Monkeys Using DNA Metabarcoding

**DOI:** 10.1002/ece3.73008

**Published:** 2026-02-03

**Authors:** Joey Felsch, Eduard Mas‐Carrió, Stéphanie Mercier, Judith Schneider, Sofia Forss, Erica Van de Waal, Luca Fumagalli

**Affiliations:** ^1^ Laboratory for Conservation Biology, Department of Ecology and Evolution University of Lausanne Switzerland; ^2^ Division of Behavioural Ecology, Institute of Ecology and Evolution University of Bern Switzerland; ^3^ Department of Evolutionary Biology and Environmental Studies University of Zurich Switzerland; ^4^ School of Life Science University of KwaZulu‐Natal Pietermaritzburg South Africa; ^5^ Department of Ecology and Evolution University of Lausanne Switzerland; ^6^ Swiss Human Institute of Forensic Taphonomy, University Centre of Legal Medicine Lausanne‐ Geneva Lausanne University Hospital and University of Lausanne Switzerland

**Keywords:** diet analysis, dietary patterns, environmental DNA, human food, primates, urbanisation

## Abstract

With increasing human domination of ecosystems, wildlife must either relocate or adapt its behaviour to anthropogenic impacts in order to survive. Vervet monkeys ( *Chlorocebus pygerythrus* ), whose natural habitats have been progressively encroached upon by urban expansion, have successfully adapted to urbanised environments because of their flexible and generalist feeding behaviour. Characterising diet composition of vervet monkeys can therefore reveal how they exploit anthropogenic resources and uncover opportunistic foraging behaviours. However, accurately determining complete diets through direct observations is challenging. In this study, we used an environmental DNA (eDNA) approach investigating the DNA mixtures present in faecal samples as a non‐invasive complementary method for assessing diet and foraging strategies. We identified the dietary components of vervet monkeys through DNA metabarcoding of 447 faecal samples collected from two monkey groups over 4 months in a semi‐urban neighbourhood in South Africa. We further compared the results with observational data on foraging to describe how vervet monkeys exploit anthropogenic resources. Subsequently, we evaluated whether dietary patterns can be distinguished between groups and within matrilineal levels. We found DNA metabarcoding data to be consistent with observational data, but the former revealed a broader diversity of consumed taxa. Additionally, we detected a difference in diet between the two investigated groups, and a tendency for similar dietary patterns among matrilineal pairs compared to other group members. Our results support the use of the DNA metabarcoding methodology, both to determine the complex diet of omnivorous species in urbanised ecosystems and to address interindividual foraging behaviours.

## Graphical abstract

We used eDNA metabarcoding of faecal samples to quantify the dietary composition of two vervet monkey populations in a semi‐urban environment in South Africa over a 4‐month period, and compared these results with visual observations. We found that both methods yielded similar results, but eDNA metabarcoding revealed a greater diversity of consumed taxa. We also detected dietary differences between the groups, as well as a similar pattern among matrilineal individuals compared to other group members.

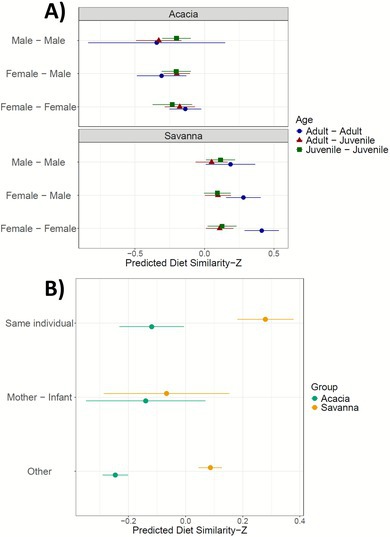

## 1 Introduction

Ecosystems are increasingly fragmented by human activities (Yang et al. 2025), often resulting in the restructuring of faunal and floral compositions (Mainwaring et al. 2024; McKinney 2002). Consequently, local species must adapt to these disturbances to survive (Johnson and Munshi‐South 2017), or relocate and, in the worst case, face extinction. For example, the red fox ( *Vulpes vulpes* ) has been actively colonising urban areas over the past few decades, facilitated in part by the availability of anthropogenic food resources (Bateman and Fleming 2012). We also know that certain primate species, such as long‐tailed macaques ( *Macaca fascicularis* ) or vervet monkeys ( *Chlorocebus pygerythrus* ), utilise suburban areas as refuges and feeding sites, enabling them to thrive in heavily urbanised landscapes (Ilham et al. 2017; Patterson et al. 2017). Such adaptations include exploiting resources of human origin, raising concern for urban wildlife management because of the dependence on anthropogenic food, which can lead to conflicts with humans (Else 1991; Mazué et al. 2023; Wimberger et al. 2010).

Animals living close to urban areas may vary in their consumption of anthropogenic food (Barrett 2005). Investigating the composition of non‐natural components of their diet could disentangle associated behavioural changes and improve conservation and management strategies (Aleixo‐Pais et al. 2023; Berger et al. 2001). However, understanding the role of consumer‐resource interactions in food webs is limited by the difficulty of precisely determining the diversity of foods consumed by animals. This challenge is particularly pronounced for generalist species, as they consume a diverse range of plant and animal species (De Barba et al. 2014; Pompanon et al. 2012). Until recently, methods for analysing diet have mainly included direct observation, microscopic examination of faeces, and protein electrophoresis of gut contents (Aleixo‐Pais et al. 2023; De Barba et al. 2014; Pompanon et al. 2012). However, these approaches have limitations in accurately characterising the full range of foods that constitute a diverse diet.

DNA metabarcoding is an increasingly used technique for studying animal diets (Glenn 2011; Pompanon et al. 2012; Shendure and Ji 2008; Taberlet et al. 2018), as it allows the parallel identification of the different taxa consumed by an animal through the DNA present in their faeces, stomach contents or regurgitations (i.e., environmental DNA). This is achieved by the simultaneous amplification of short DNA metabarcodes using universal primers, which are taxonomically resolutive (Deagle et al. 2009, 2013; Taberlet et al. 2012; Valentini, Miquel, et al. 2009; Valentini, Pompanon, and Taberlet 2009). DNA metabarcoding provides a broader taxonomic coverage and resolution than traditional observational methods for dietary analysis (Brun et al. 2022) and can be employed to predict spatial and temporal biodiversity patterns in ecosystems (Mas‐Carrió et al. 2022; Thomsen and Willerslev 2015). Hence, this technique can offer a more accurate assessment of human‐derived components in the diet, improving our understanding of anthropogenic influences on feeding behaviour. These molecular approaches are of considerable interest for the analysis of complex diets (Deagle et al. 2009; Deagle and Tollit 2007), as they do not require prior knowledge of the potential food items consumed by animals in the habitat they occupy (De Barba et al. 2014).

**FIGURE 1:**
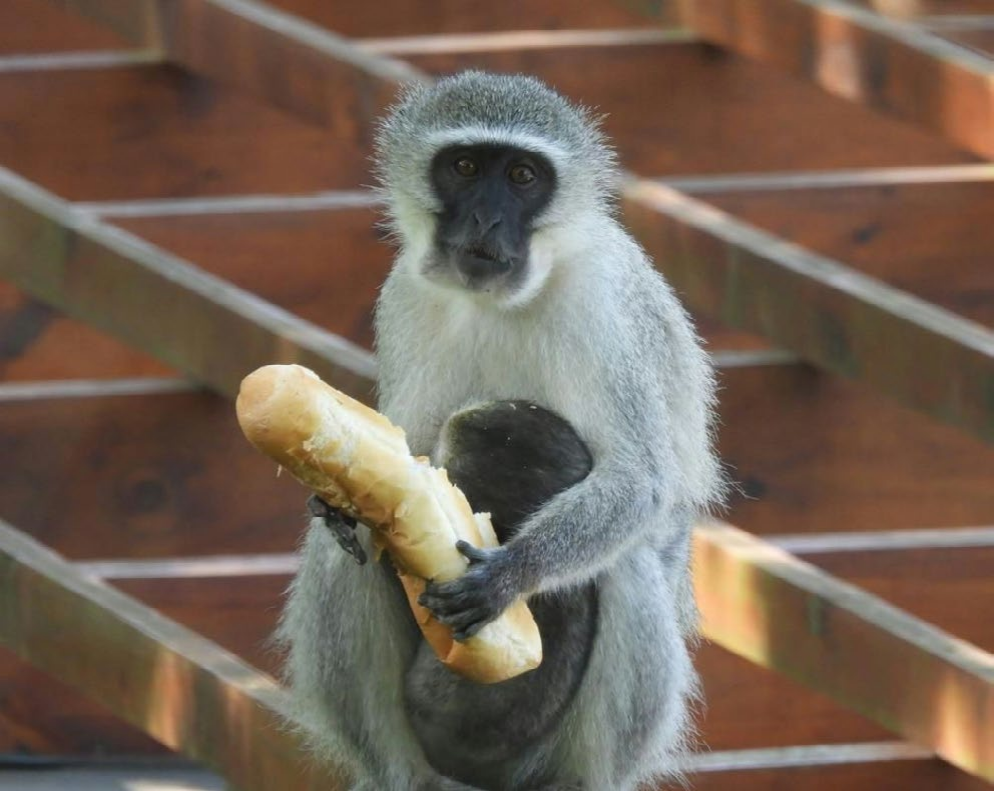
A mother vervet monkey ( *Chlorocebus pygerythrus* ) carrying her infant and bread. Picture by Adrian McConnell.

Vervet monkeys have a large distribution area in eastern Africa, ranging from Ethiopia to South Africa (Wilson and Reeder 2005). During recent decades, their range has expanded considerably in urbanised areas of South Africa (Whittaker et al. 2013), partly because of increased habitat fragmentation and transformation (Strum 2010). As generalist primates, vervet monkeys are pre‐adapted to exploit diverse food sources (Barrett 2005; Basckin and Krige 1973; Isbell et al. 1998; Johnson‐Ulrich and Forss 2025), which may explain their ability to thrive in human‐altered landscapes (Albert et al. 2014). Several studies have shown that vervet monkeys can take advantage of human‐modified habitats and the unnatural food they provide (Horrocks and Baulu 1994; Takahashi 2018), as human food has a high energy content (Hoffman and O'Riain 2012; Thatcher et al. 2019; Figure 1). Thus, vervet monkeys can serve as a model species for understanding the persistence of wildlife in the ongoing transformation of the human‐dominated landscape (Ellington et al. 2024; Foard et al. 1994; Patterson et al. 2018; Robira et al. 2025).

Vervet monkeys exhibit plastic feeding behaviour according to resource availability, nutritional requirements, and environmental conditions, which can lead to intraspecific diet variation (Kratina et al. 2012; Williams et al. 2004). Such differences may be rooted in the species' ability to acquire knowledge about what to eat through social learning (Agostini and Visalberghi 2005; Hoppitt and Laland 2013; Jaeggi et al. 2010; Schiel and Huber 2006; van de Waal et al. 2012, 2013, 2014). Thus, leveraging social information from conspecifics may facilitate adaptation to the local environment (Rendell et al. 2010). Different studies have demonstrated social transmission in vervet monkeys, leading to dietary habits at the group level (Canteloup et al. 2020; Dongre et al. 2024; Tournier et al. 2014; van de Waal et al. 2012, 2013). However, although it is possible that there are differences in overall diet composition between groups of vervet monkeys, it is not yet clear whether these differences are due to social learning clustering into local traditions or emerge from ecological circumstances (Schneider et al. 2023; Tournier et al. 2014). Furthermore, social learning can canalise into traditions within the maternal lineage level rather than spreading out at the group level (van de Waal et al. 2012, 2014). Therefore, studying the diets of vervet monkeys in an urbanised environment can help determine whether inter‐ and intra‐group traditions emerge in these populations.

In this study, we performed a methodological comparison between observational and DNA metabarcoding data from faecal samples of two groups of vervet monkeys in a semi‐urban environment at the Simbithi Eco‐Estate in Ballito, South Africa. We described their diets using the two methodologies and quantified the proportion of diet derived from anthropogenic resources. We also aimed to elucidate whether social variations can be detected, such as dietary differences between groups or individuals. We hypothesised (i) that molecular methods will provide broader taxonomic coverage and better resolution than observational data, (ii) that dietary differences would emerge among social groups as a result of distinct foraging behaviours, with access to anthropogenic food sources allowing a greater diversity of resources and (iii) that individuals with closer relationships such as mother‐offspring pairs will have more similar diets because of predicted social transmission of dietary preferences (van de Waal et al. 2013). Given the rapid changes in habitats, assessing the relative contribution of different foods in the diet is important to better understand the impact of generalist species within an urbanised ecosystem.

## 2 Materials and Methods

### 2.1 Study Area

Data for this study were collected at the Urban Vervet Project, located within the Simbithi Eco‐Estate, a 4.7 km^2^ private gated community in Ballito, north of Durban, KwaZulu‐Natal, South Africa (29.5140° S, 31.2197° E). In this habitat, spaces between residences are landscaped into small coastal human‐engineered forest areas, with several ponds and marshes distributed throughout the estate. Although the estate is surrounded by a large electric barrier, it does not prevent the monkeys from crossing it. Vegetative corridors are present between houses to facilitate wildlife movement. Only indigenous plant species may be planted on the estate, whereas exotic species should be maintained in pots. Trash containers occur outside houses and can be opened by the monkeys, unless a locking mechanism is installed.

### 2.2 Study Population

**FIGURE 2:**
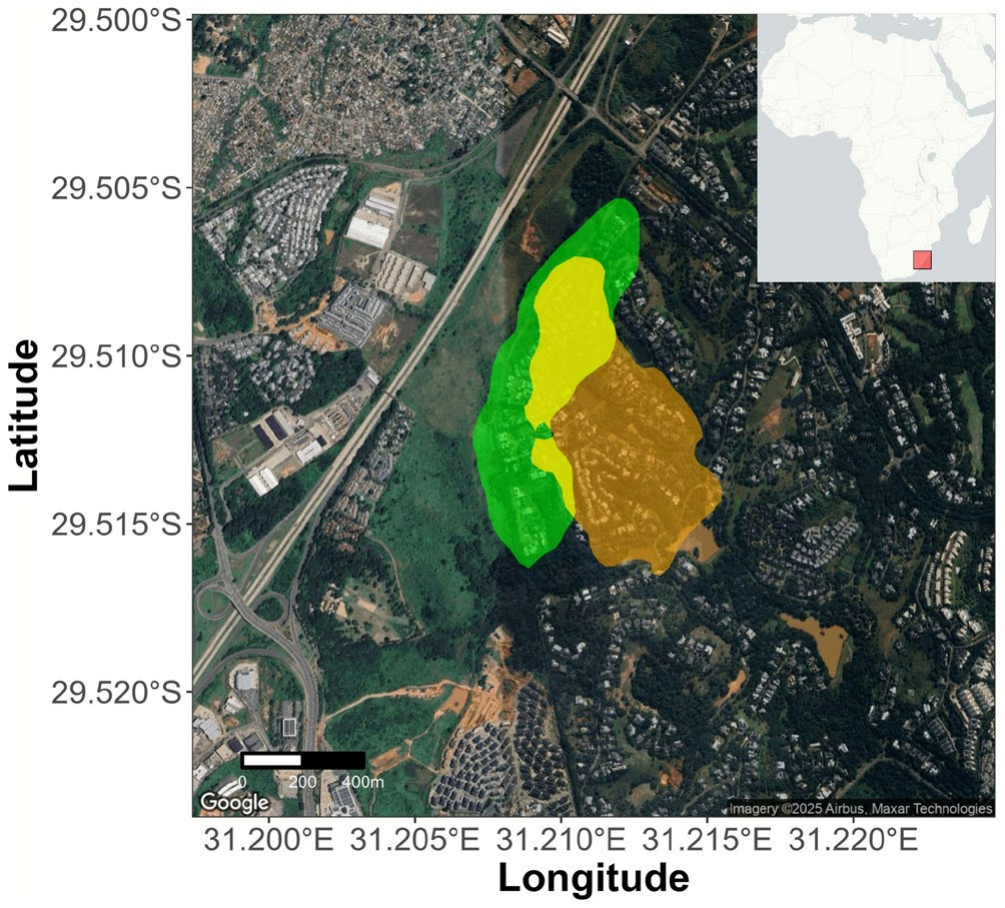
Ranges of the two monkey groups in Simbithi Eco‐Estate. Ranges are represented by the coloured polygons (green for the Acacia group, orange for the Savanna group and yellow for the overlap between the groups).

We studied two neighbouring groups within this study site: Acacia and Savanna. Although group size varied over the study period (because of births, deaths, and dispersals), data were consistently collected from 20 individuals in Acacia (adults: 1 male, 6 females; juveniles: 8 males, 5 females) and 23 in Savanna (adults: 3 males, 6 females; juveniles: 7 males, 7 females; Table S1). For each group, 95% of the habitat utilisation distribution was calculated, estimated from GPS records obtained between 15 August 2023 and 22 December 2023 (Acacia group = 128 tracking days, totalling 343 h; Savanna group = 127 tracking days, totalling 494 h). GPS locations were obtained by the observers using a handheld GPS (Blackview device; Gaia GPS 2024), which was set up to record locations continuously (i.e., as soon as movement exceeding the GPS error threshold was detected). To facilitate processing, we transformed the GPS tracks at 30 s intervals if they were not separated by more than 120 min. We estimated groups' utilisation distributions using Brownian‐based movement kernels (Benhamou 2011) with the “BRB” function of the *adehabitatHR* package (Calenge and Fortmann‐Roe 2023). For this, we considered movement to have occurred when the GPS locations were more than 15 m apart (*L*_*min*_). A Brownian bridge was considered only between locations separated by no more than 120 min (*T*_*max*_), and we considered a smoothing of 30 m (*h*_*min*_). The coefficient of diffusion was estimated on the basis of the movement tracks using the “BRB.D” function. The ranging areas for the two groups (taking topography into account) were 34.8 ha (Acacia) and 41.0 ha (Savanna). Topography was extracted using the “get_elev_raster” function of the *elevatr* package (Hollister 2023) from Amazon Web Services Terrain Tiles. The volumetric overlap between the two home ranges (i.e., the Bhattacharyya coefficient) was 0.42 (Figure 2).

Within both groups, each monkey was identified on the basis of distinctive bodily and facial features (e.g., scars, colours, and shapes). All observers involved in this study passed an identification test to ensure individual recognition, as well as an inter‐observer reliability test conducted with the experienced onsite manager. A minimum Cohen's Kappa value of 0.8 was required for validating this test. An individual was classified as an adult if it had given birth for the first time (females), or had left the natal group (males), and as an infant if it was less than 1 year old. Infants and all other individuals except adults were classified as juveniles in the analysis.

### 2.3 Faecal Samples

Faecal samples were collected opportunistically from 15 August 2023 to 22 December 2023. In total, 447 faecal samples from 43 individuals were collected during the study period (Tables S2 and S3). Sample collection was performed only after individual identification during observed defecation events, ensuring accurate assignment of samples to individuals. Faecal samples were collected within 15 min to limit the impact of external contaminants (De Barba et al. 2014; Oehm et al. 2011). Faecal samples were not re‐collected from individuals who had already been sampled on the same day. Between 0 and 6 samples were collected per month for each monkey (mean = 2.08). Approximately 0.5 cm^3^ of the internal part of the faeces was collected using gloves and plastic sticks into 20 mL high‐density polyethylene (HDPE) scintillation vials (Carl Roth GmbH) filled to about one‐third with 95% ethanol. After 24 to 48 h, the ethanol was replaced with silica gel beads, and the dried faecal samples were stored until DNA extraction (Brun et al. 2022).

### 2.4 Observational Data

Observational data were collected using Focal Follow animal sampling (Altmann 1974). During a focal, individuals were followed for 20 min, and their actions and interactions (e.g., movement, feeding, social interactions, vocalisations) were recorded in a continuous way. If the focal individual was out of sight for a total of more than 5 min, the focal was aborted. Individuals were opportunistically chosen during three different time slots of the day (morning, noon, and afternoon) to achieve at least one successful focal sample per individual per time slot each month to have a balanced dataset through the day and a similar amount of observation for each monkey (Brun et al. 2022). Monkeys were followed 5 days a week by researchers for about 8 h a day ranging from sunrise to sunset on either morning or afternoon shifts. Data were collected using the *CyberTracker* application (CyberTracker 2024) on Blackview devices. From this data, we extracted information related to feeding observations concerning both plant material and anthropogenic resources (e.g., processed foods, non‐native fruits). Observational data on the 43 studied monkeys were used to compare the results obtained with DNA metabarcoding methods. Although observational data were collected throughout the year, we used only the data corresponding to the time period of faecal sample collection to ensure a direct comparison between the two methods.

### 2.5 Faecal DNA Extraction

DNA was extracted from faecal samples using a phosphate buffer‐based approach (Taberlet et al. 2018), on the basis of the protocol from the NucleoSpin Soil Kit (Macherey‐Nagel), with the following modifications. The faeces in the scintillation vials were directly transferred to 2 mL Eppendorf tubes with 1.3 mL of phosphate buffer. The samples were placed on a tube rotator for 15 min to facilitate DNA absorption and later homogenised by vortexing. The samples were then centrifuged for 5 min. The subsequent steps of the extraction required the use of QIAvac technology (Qiagen). DNA extractions were performed in a dedicated laboratory designed for handling low‐template DNA samples (Laboratory for Conservation Biology, University of Lausanne). For subsequent analyses, all DNA extracts were diluted fivefold.

### 2.6 DNA Metabarcoding Assay

DNA extracts were amplified in triplicate using two sets of primers, one for plants and one for vertebrates. Although vervet monkeys may also consume other items (i.e., invertebrates and fungi), our analysis was restricted to the two former taxa, as they best align with the principal dietary habits of humans. The first primer pair (*Sper01*; forward: 5′‐GGGCAATCCTGAGCCAA‐3′; reverse: 5′‐CCATTGAGTCTCTGCACCTATC‐3′) targets plant components of the diet by amplifying the P6 loop of the *trnL* (UAA) intron of chloroplast DNA (10–220 bp; Taberlet et al. 2018). The second primer pair (*Vert01*; forward: 5′‐TTAGATACCCCACTATGC‐3′; reverse: 5′‐TAGAACAGGCTCCTCTAG‐3′) amplifies a fragment of the mitochondrial rDNA 16S, and is highly specific for vertebrates (56–132 bp; Taberlet et al. 2018). For the latter, a blocking oligonucleotide (5′‐CTATGCTTAGCCCTAAACCTCAGTAGTTAAACCAACAAAACTACT‐C3‐3′) was added to specifically inhibit the amplification of vervet monkey DNA. PCR primers included 5′ tags, consisting of an 8‐nucleotide sequence with at least 3 nucleotide differences between each tag, used for assigning sequences to their respective sample. PCR reactions were performed in a final volume of 20 μL in 96‐well plates. For the *Sper01* primers, the mix contained: 1 × AmpliTaq Gold 360 (Applied Biosystems), 0.5 μM of forward and reverse primers, 0.16 mg/mL of Bovine Serum Albumin (BSA; Roche Diagnostics), and 2 μL of template DNA. For the *Vert01* primers, the mix contained 1 × AmpliTaq Gold 360 (Applied Biosystems), 0.2 μM of forward and reverse primers, 0.16 mg/mL of Bovine Serum Albumin (BSA; Roche Diagnostics), 2 μM of blocking oligonucleotide, and 2 μL of template DNA.

PCR cycling conditions were 10 min at 95°C, followed by 40 cycles of 30 s at 95°C, 30 s at 52°C or 49°C (for *Sper01* and *Vert01*, respectively), and 60 s at 72°C, with a final extension of 7 min at 72°C and 10 min at 4°C. For each PCR plate, a negative extraction control, a negative PCR control (ultrapure water), positive controls, and blanks were included. Positive controls contained DNA of known concentration from plant or vertebrate species not expected at the study site, serving to validate the amplification success (Table S5). Amplification success was verified by 2% agarose gel electrophoresis for a subset of samples. Finally, PCR products for each primer pair were pooled for library preparation.

### 2.7 DNA Sequencing

Amplicons were purified using the MinElute PCR Purification Kit (Qiagen) and quantified with a Qubit 4 Fluorometer (Invitrogen). Fragment sizes and relative abundance of the amplicons were quantified using a Fragment Analyzer (Agilent Technologies). Libraries were then prepared using a protocol on the basis of the TagSteady approach (Carøe and Bohmann 2020). Library quantification was performed using a qPCR Real‐Time System (Bio‐Rad) to ensure accurate quantification. Following quantification, the size of the DNA fragments in the libraries was measured again using a Fragment Analyzer. Sequencing was performed on an Illumina MiniSeq System, using the Mid Output Kit generating eight million 150 bp paired‐end reads.

### 2.8 Local Plant Database

On the basis of information from local botanical experts, we established a list of plants that are present in the study area and potentially consumed by the vervet monkeys. For each of these plants, we verified their presence in GenBank. Those that were missing or had low alignment scores to the targeted metabarcode (Identity < 98%, Coverage < 70%, *e*‐value > 5) were identified morphologically in the field and collected for later sequencing, to create a custom database for the missing elements. In total, 37 plant species were sampled (Table S4). For each species, two pieces of one cm^2^ leaf fragments were dried with silica gel beads in 20 mL HDPE scintillation vials (Carl Roth GmbH) and stored until DNA extraction, except for nine succulent plant species, which were preserved in 95% ethanol. DNA extraction was performed using the DNeasy Plant Mini Kit (Qiagen). PCR reactions were then carried out on the entire chloroplast *trn*L (UAA) intron region using primers *c* and *d* (Taberlet et al. 2007). They were performed in a total volume of 25 μL, containing: 1 × PCR Gold Buffer (Thermo Fisher Scientific), 2 mM of MgCl_2_, 0.2 mM of dNTPs, 0.5 μM of forward and reverse primers, 1 U of AmpliTaq Gold 360 (Applied Biosystems), and 2 μL of template DNA diluted 250‐fold. PCR cycling conditions consisted of an initial denaturation of 5 min at 95°C followed by 45 cycles of 30 s at 95°C, 30 s at 5°C and 60 s at 72°C, with a final elongation of 5 min at 72°C. Subsequently, purification and Sanger sequencing were conducted at Microsynth AG (Balgach, Switzerland). The sequences were aligned using MEGA11 v. 11.0.13 (Tamura et al. 2021; Figure S1).

### 2.9 Bioinformatics Processing

Sequencing results from each library were handled separately using the *OBITools* package (Boyer et al. 2016). First, forward and reverse reads were assembled with a minimum quality score of 40 and assigned to each corresponding sample on the basis of unique tags and primers. Identical sequences were then clustered. Sequences that could not be aligned, as well as those with fewer than 10 reads per library or those not meeting the appropriate primer length, were removed. After correcting for PCR/sequencing errors, remaining clusters were reduced on the basis of a 97% similarity threshold using the *sumaclust* algorithm (Mercier et al. 2013).

Taxonomic assignment of sequences was based on three sources: the local plant database we created (see above), and two databases generated from in silico PCR simulations using our two sets of primers with a 95% similarity threshold. These simulations were queried against the GenBank database maintained by the National Center for Biotechnology Information (NCBI) using the ecoPCR software (Ficetola et al. 2010). Additionally, each operational taxonomic unit (OTU) identified for *Sper01* and *Vert01* was manually verified using BLAST on GenBank to confirm the results. Further sequence cleaning and analyses were conducted using the *metabaR* package (Zinger et al. 2021). In addition, because experiments involving peanuts as food rewards were conducted during the study period, we removed all DNA reads assigned to the genus *Arachis* in the faecal samples collected the day after these experiments took place.

### 2.10 Data Analyses

Data analyses were conducted using R (version 4.2.1; R Core Team 2022) via RStudio (version 2023.12.1). Retained sequences were transformed to relative read abundance (RRA), which represents the percentage of presence of a taxon in a sample calculated from its read count. Taxa present in only one of the three replicates were removed from the analyses, and the mean RRA was used to merge replicates into a single dataset. For the observational data, we considered the number of seconds of active feeding as a measure of relative abundance to make comparisons (Gérard et al. 2024). A logit transformation was applied to the proportions of the sum of RRA and of the seconds to achieve normality:

$$\text{logit}\left(p\right)=\log\left(\frac{p}{1-p}\right)$$

For the comparative analyses, we considered only taxa identified at the genus level or higher, as the observational data were only taxonomically precise up to the genus level. The results were visualised as a Venn diagram illustrating the overlap and differences in species consumed detected by the two methods. Observational data already distinguish between anthropogenic and natural food sources. As this distinction was not provided by the faecal data, a score representing the proportion of anthropogenic food was assigned to each taxon on the basis of field observations and existing knowledge. A score of 0 was assigned to taxa confirmed as natural resources, a score of 1 to taxa confirmed as anthropogenic food, and a score of 0.5 to taxa that could plausibly be either a natural resource or anthropogenic food in the study area (e.g., an avocado, which could be obtained from houses or found naturally in trees; Table S6). On the basis of these scores, a percentage was calculated per sample, where the sum of each taxon's score multiplied by its RRA was then divided by the sum of RRAs in that sample. These percentages were then visualised using a density plot. To examine potential inter‐ and intragroup differences, we then aimed to determine the diet of vervet monkeys based solely on faecal samples and to assess differences related to age, sex, or social affiliation.

Dissimilarity between faecal samples, on the basis of the RRA of consumed species, was calculated using Bray‐Curtis distance, with the *vegan* package (Oksanen et al. 2007). We visualised dissimilarities between groups on a Non‐metric MultiDimensional Scaling (NMDS). Using the *performance* package (Lüdecke et al. 2021), we assessed the distribution of the standardised similarity of our data, which displayed a normal distribution (random forests model on the basis of residuals). With this information, we performed Generalised Linear Mixed Models (GLMMs) with a Gaussian distribution family to analyse the impact of social variables on the standardised similarity. The models were then compared using several performance indices (Akaike Information Criterion AIC, Bayesian Information Criterion BIC, Root Mean Squared Error RMSE, and the coefficient of determination R^2^). Ultimately, we retained the two following statistical models:

(1) $$\begin{array}{c} \text{Similarity}-Z\sim \text{Group}*\;\mathrm{Sex}*\;\mathrm{Age}+\left(1|\text{Individuals}\right)+\\ \left(1|\text{Date}\right)+\left(1|\text{Relationship}\right) \end{array}$$

(2) $$\text{Similarity}-Z\sim \text{Group}*\;\text{Relationship}+\left(1|\text{Individuals}\right)+\left(1|\text{Date}\right)$$

We considered for these models only two age categories: adult or juvenile. The relationship between individuals included three categories: comparing an individual's sample with another sample from the same individual (Same individual), comparing a sample from an infant with that of its mother (Mother—Infant), or comparing any other relationship (Other).

## 3 Results

**FIGURE 3:**
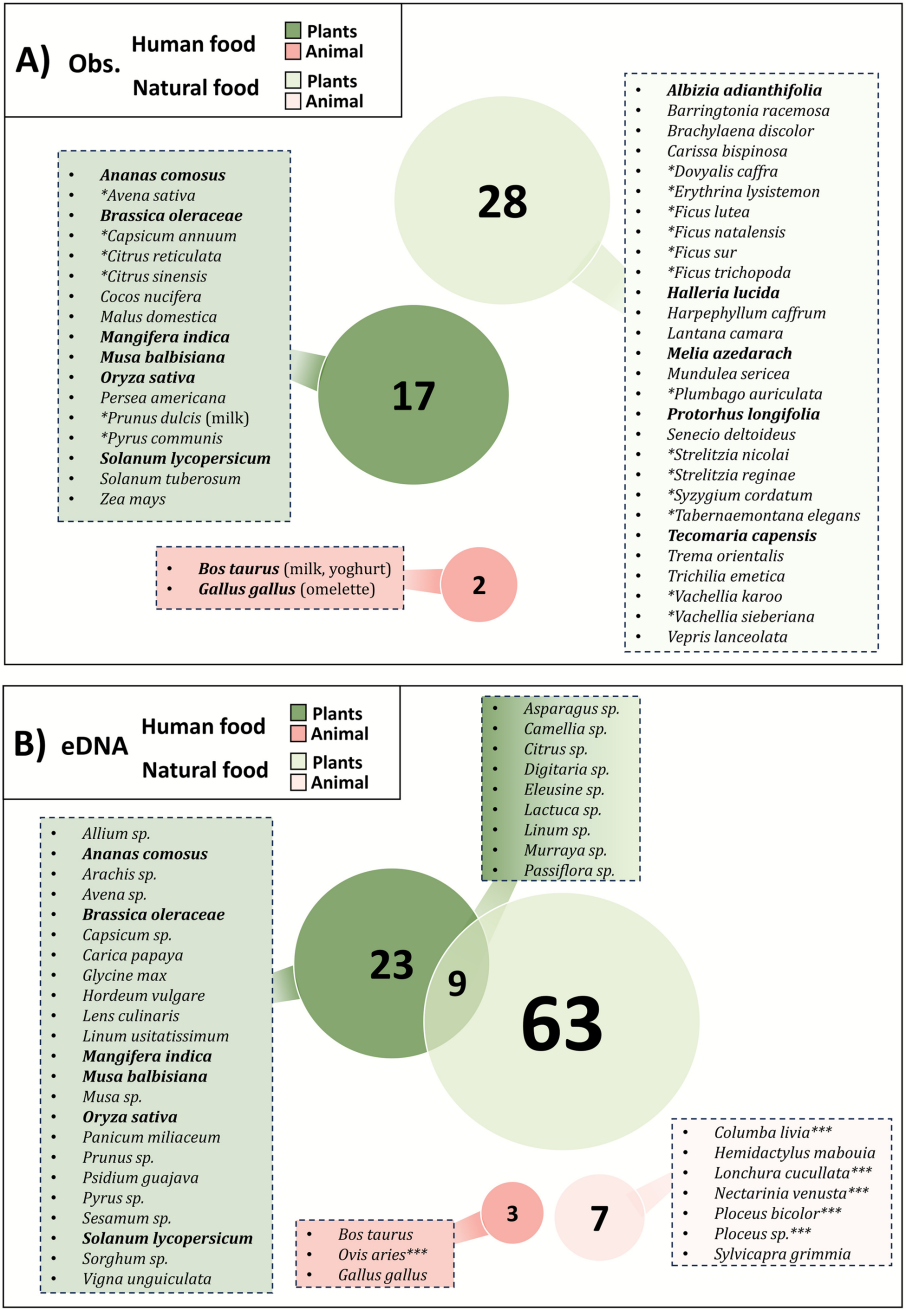
Venn diagram of plant taxa (in green) and vertebrate taxa (in red) at the genus and species taxonomic levels, for anthropogenic food (dark colour) and natural food (light colour) detected by (A) observational data and (B) eDNA. Species marked with an asterisk (*) correspond to genera detected through eDNA data, whereas those marked with three asterisks (***) indicate species that may correspond to observational records. Species names in bold appear in both datasets, and the number in the circle indicates the total number of species in that category.

For the observational data, a total of 640 focal samplings (Table S2) allowed us to record 831 observations of natural plant consumption by the monkeys (28 different species) that could be identified by the observers, 313 observations of unknown natural plant species, and 55 observations of anthropogenic food consumption, although detailed information about the specific food items was not always available (Figure 3A).

For the 447 faecal samples analysed, a total of 6,897,325 reads were retained for the primer *Sper01*, representing 144 distinct plant taxa with a resolution of 38 species, 57 genera, six tribes, seven subfamilies, 32 families, three orders, and one subclass. For the primer *Vert01*, 15,098 reads were obtained, corresponding to 12 vertebrate taxa with a resolution of nine species, one genus, one family, and one order (Table S7). The identified taxa included various plant growth forms such as trees, shrubs, succulents, grasses, and domestic crops, as well as vertebrates like wild birds, reptiles, and livestock.

The proportion of vertebrate DNA in the vervet monkeys' diet was lower than that of plant DNA, both in terms of quantity and diversity. However, the provenance (anthropogenic or natural) of the plant taxa found in the faecal samples could not always be determined (Figure 3B and Table S6). The most frequently consumed vertebrate was chicken ( *Gallus gallus* ), and it was one of the few identified vertebrate taxa of anthropogenic origin, along with beef ( *Bos taurus* ) and sheep ( *Ovis aries* ). The remaining consumed vertebrates found in faecal samples consisted of local bird species ( *Columba livia* , *Lonchura cucullata* , *Nectarinia venusta* , *Ploceus bicolor* ), a lizard species ( *Hemidactylus mabouia* ), and a small antelope, the common duiker ( *Sylvicapra grimmia* ). Out of the 447 faecal samples collected, only 34 contained vertebrate DNA (7.6%), with the majority belonging to the Savanna group (26 out of 34 faecal samples, 76.5%). Among plants, the total relative read abundance was predominantly distributed among five taxa: *Ficus* sp. (29.4%), *Erythrina* sp. (17.1%), *Strelitzia* sp. (13.4%), Fabaceae (7.6%), and *Melia azedarach* (5.6%; Figure S2 andTable S6).

Out of the 37 species collected for the local plant database, 28 provided a successful result after sequencing. Only three taxa were detected, corresponding to one species and two genera, as some species produced similar sequences. These sequences accounted for 1,211,848 reads out of the total number (17.57%), and two of these taxa could not be assigned using the global database (Table S4).

### 3.1 Comparison of Observational and Faecal Data

**FIGURE 4:**
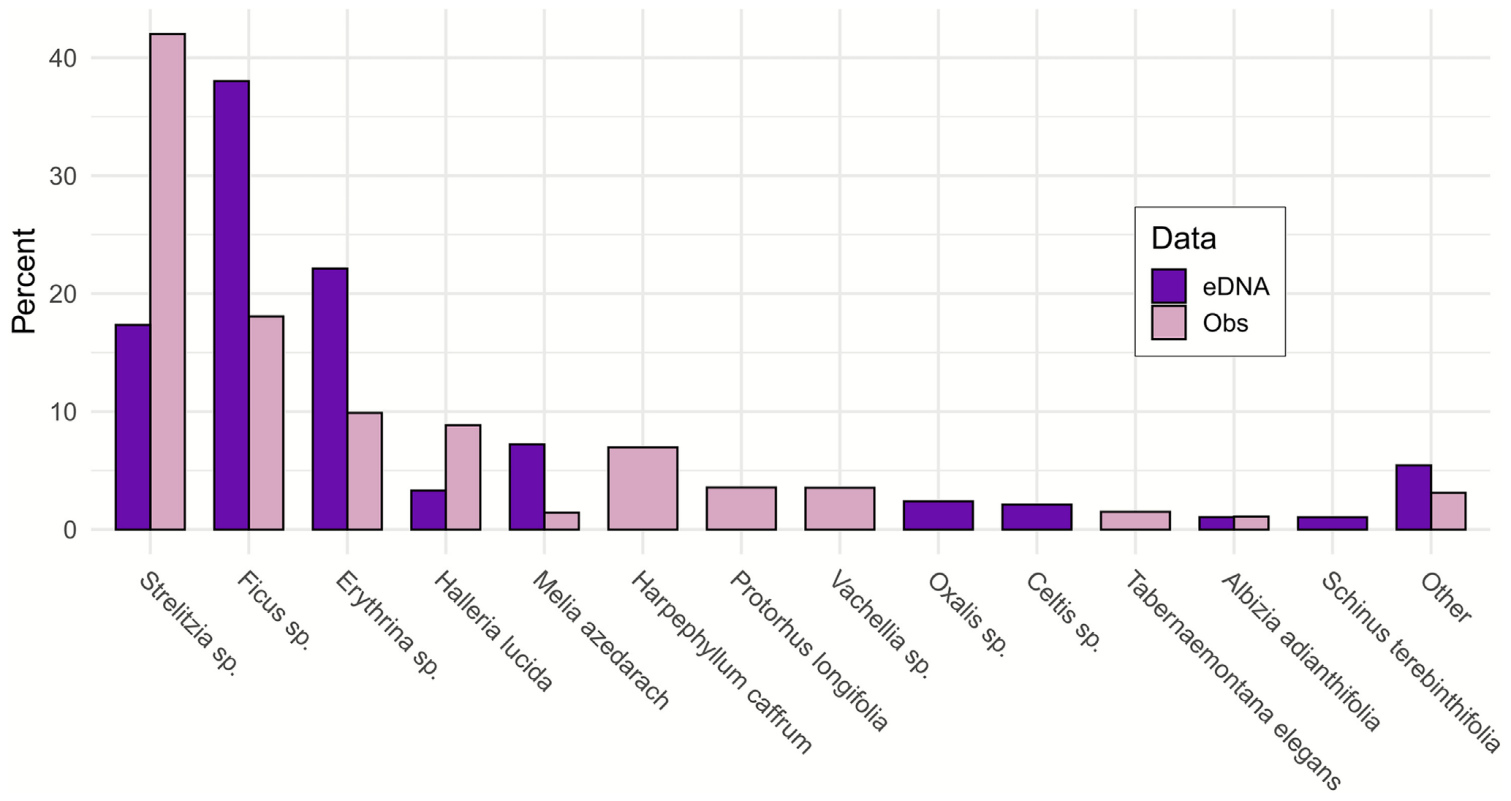
Percentage of the most abundant consumed plants from observational and faecal data at the genus level during the period of data collection. The percentage of faecal data was based on RRA, whereas the percentage of observational data was based on the number of seconds a plant was observed being eaten by a monkey.

The observational data for natural plant resources included 32 different taxa, comprising 28 species and four genera (Table S7). We visualised the most abundant plant species to provide an overview of the main dietary components (Figure 4). All the taxa below 1% were grouped as “Other”. Six of these taxa were shared between the two datasets, with the majority of the diet represented by the same three taxa (*Strelitzia* sp., *Ficus* sp., and *Erythrina* sp.).

**FIGURE 5:**
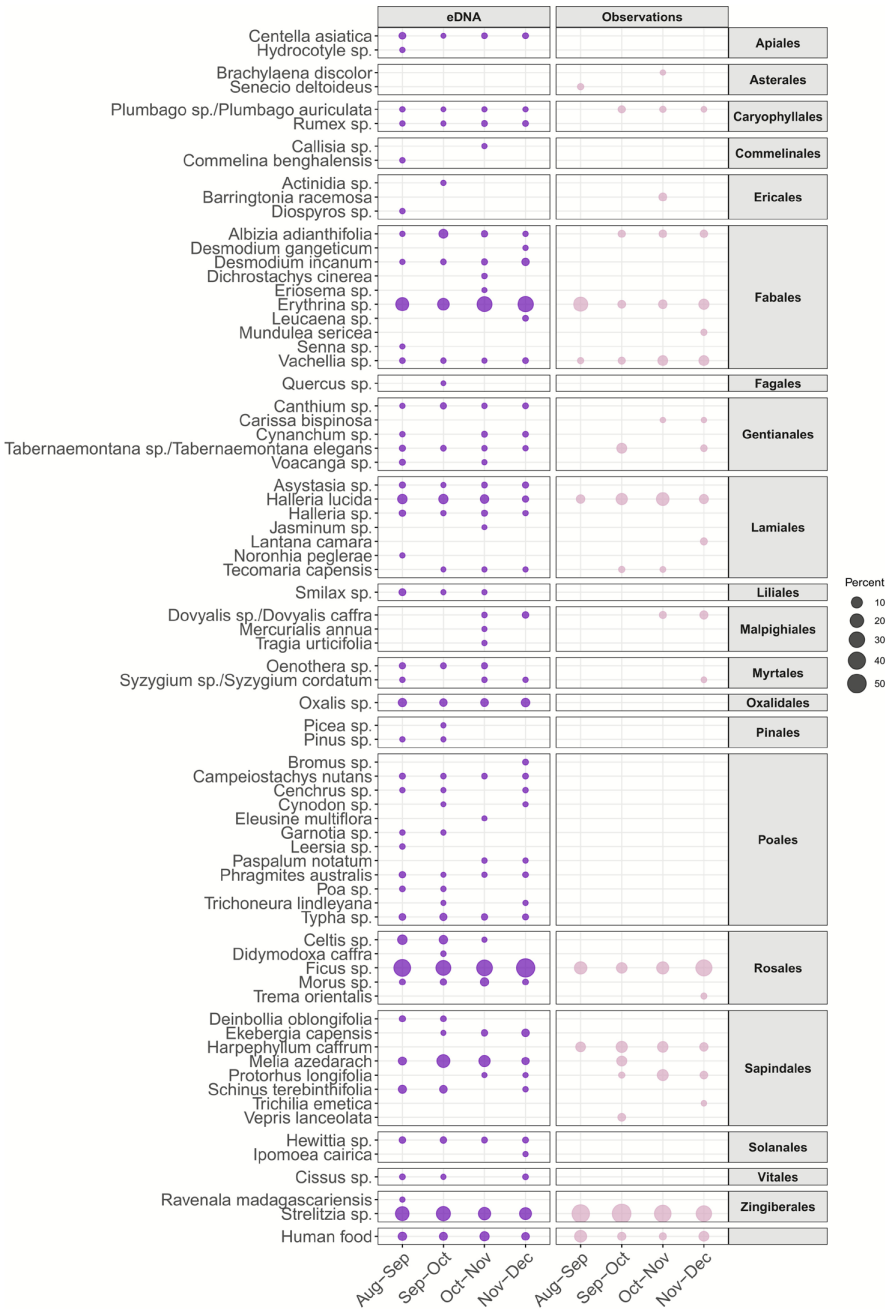
Percentage per 1‐month period (alternatively 33 and 32 days) for each taxon at a taxonomic resolution of genus and anthropogenic food for faecal and observational datasets.

We compared monthly consumption percentages for each genus and species across both datasets. Although monthly differences in diet composition were statistically significant (PERMANOVA, *R* ^*2*^ = 0.0616, *p‐value* = 0.001), these variations were not attributable to changes in the consumption of anthropogenic food, which remained relatively constant over time. Only 13 taxa matched the sampled faecal and observational data (Figure 5), limiting direct comparison. Among these, no major differences in proportions were detected across periods. Several orders were absent from the observational data (e.g., Poales), whereas only Asterales appeared in observational data and not in faecal samples.

**FIGURE 6:**
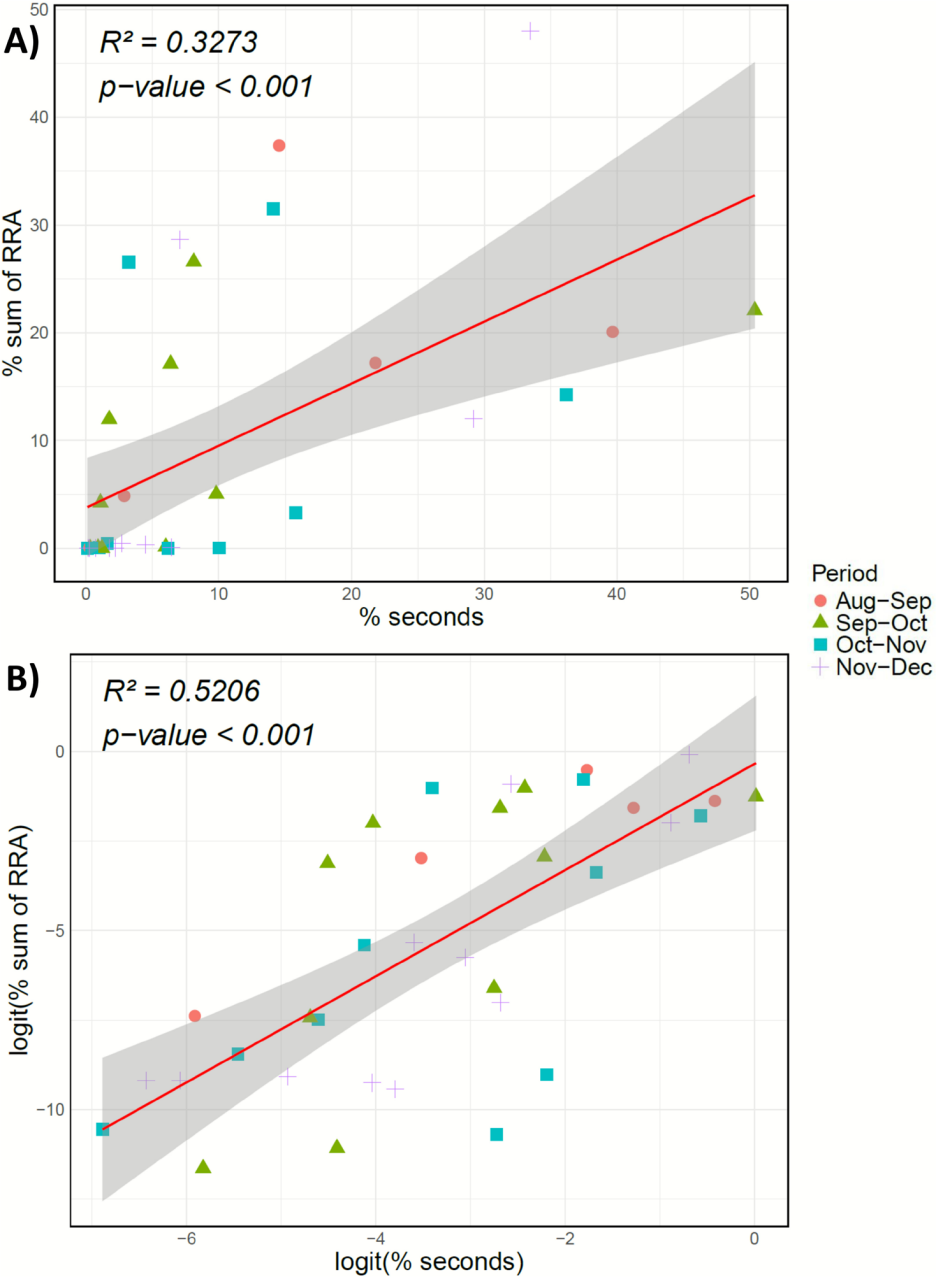
(A) Linear regression between the observational and faecal data based on the percentage of the sum of RRA and the percentage of seconds the plant was consumed during focal follows for the common plants found in both data and (B) with logit transformation.

As a next step, we fitted a linear regression model, using the 13 most common taxa between the two datasets on the basis of R^2^ and *p*‐value to test this correlation (Figure 6A). To prevent the results from being biased by high‐value points, we applied a logit transformation to the data and performed the linear regression model again (Figure 6B). The correlation coefficients for the logit transformation model indicate a significant positive relationship between the two datasets (*R* ^*2*^ = 0.5206, *p‐value* = 6.858e‐07).

**FIGURE 7:**
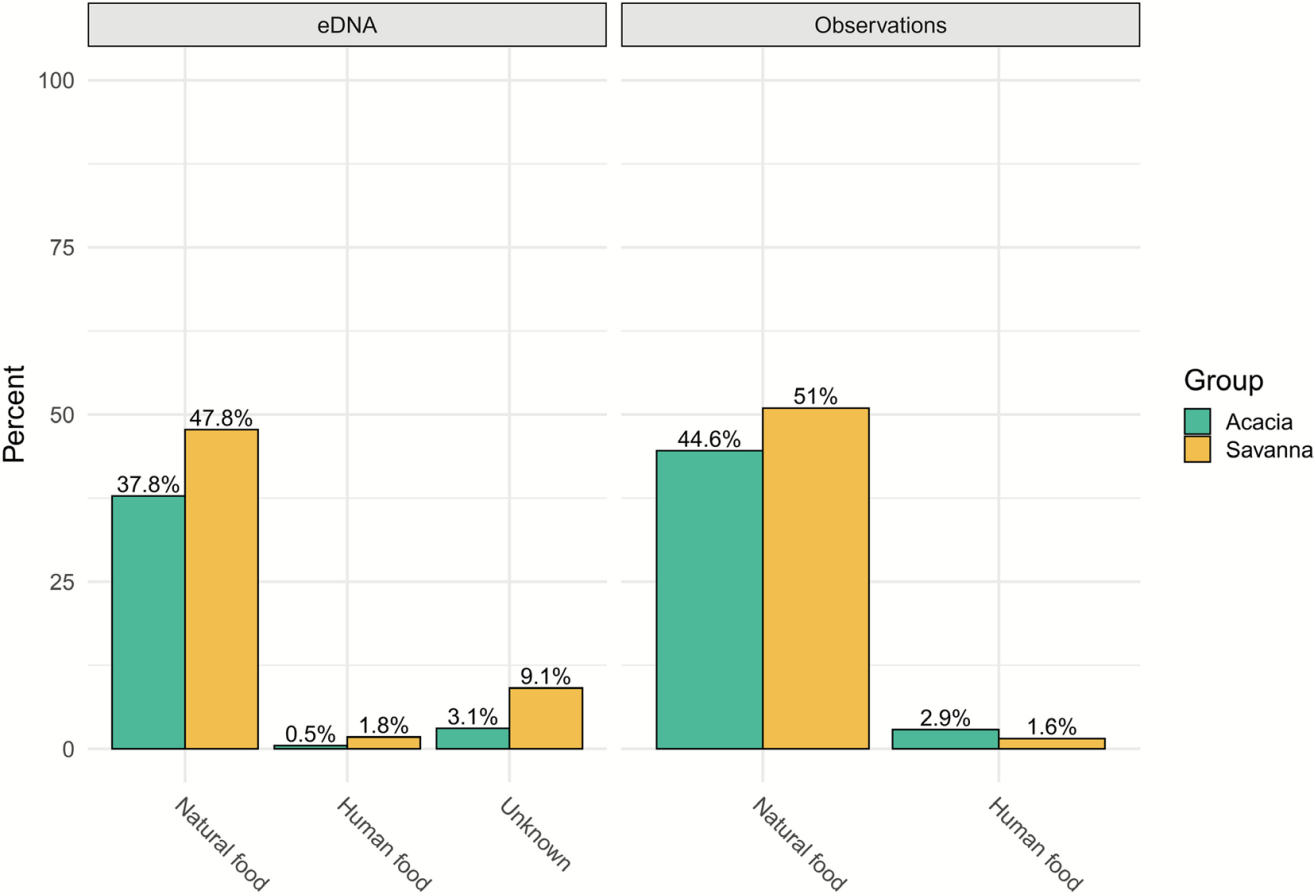
Percentage of natural and anthropogenic food, on the basis of the sum of RRA for faecal data (left) and on time for observational data (right), for the two monkey groups, Acacia (green) and Savanna (yellow). The percentages add up to 100% for eDNA and to 100% for observations.

We also aimed to determine the proportion of natural versus anthropogenic food detected in the two datasets. As illustrated in Figure 7, the proportion of anthropogenic food in the observational data was 4.5% and was mainly eaten by the individuals of the Acacia group. For the faecal data, at least 2.3% of the monkeys' diet was confidently attributed to anthropogenic food. However, 30 taxa (Table S6) were of uncertain origin when collected through faecal samples and represented a notable portion of the global diet (12.2%). In both cases, and contrary to the observational data, the proportion of anthropogenic food was at least three times higher in the Savanna group than in the Acacia group.

### 3.2 Dietary Differences Between Monkey Groups

**FIGURE 8:**
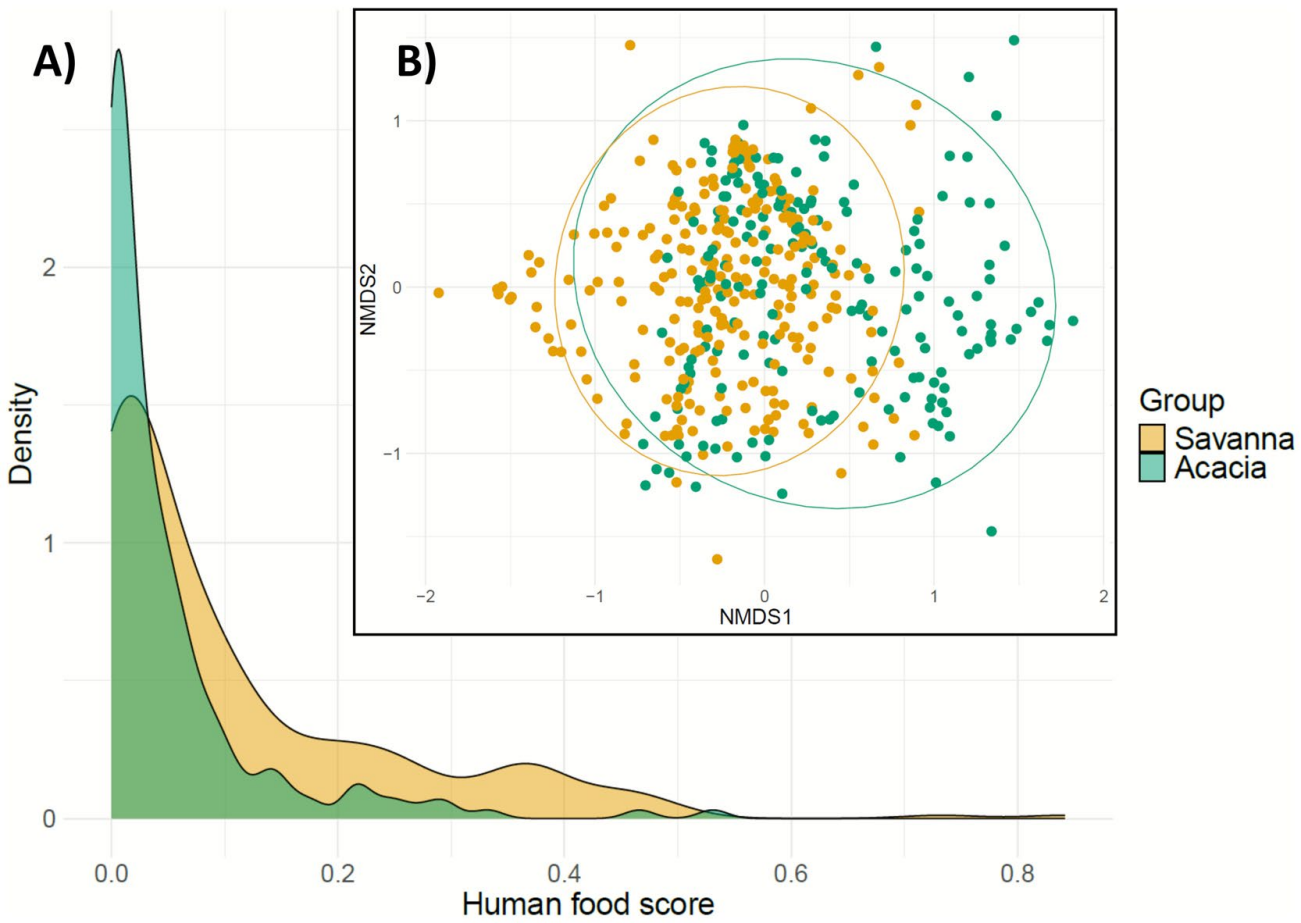
(A) Density of the percentage of anthropogenic food consumed for the two monkey groups, Savanna and Acacia, on the basis of the scores established for each taxon. A value of 0 represented natural food, a value of 1 anthropogenic food and a value of 0.5 food that could be either natural or human. (B) NMDS between faecal samples of Acacia and Savanna. Each dot represents a sample, and the closer the dots are, the more similar they are. The ellipses represent the 95% confidence intervals per group.

The eDNA data showed that the Savanna group consumed a higher proportion of anthropogenic food compared to the Acacia group (Figure 8A). Next, we performed a NMDS analysis (stress = 0.24) for each sample (RRA; Figure 8B). Although a clear overlap is evident in the 95% confidence ellipses, the overlap is not complete.

**FIGURE 9:**
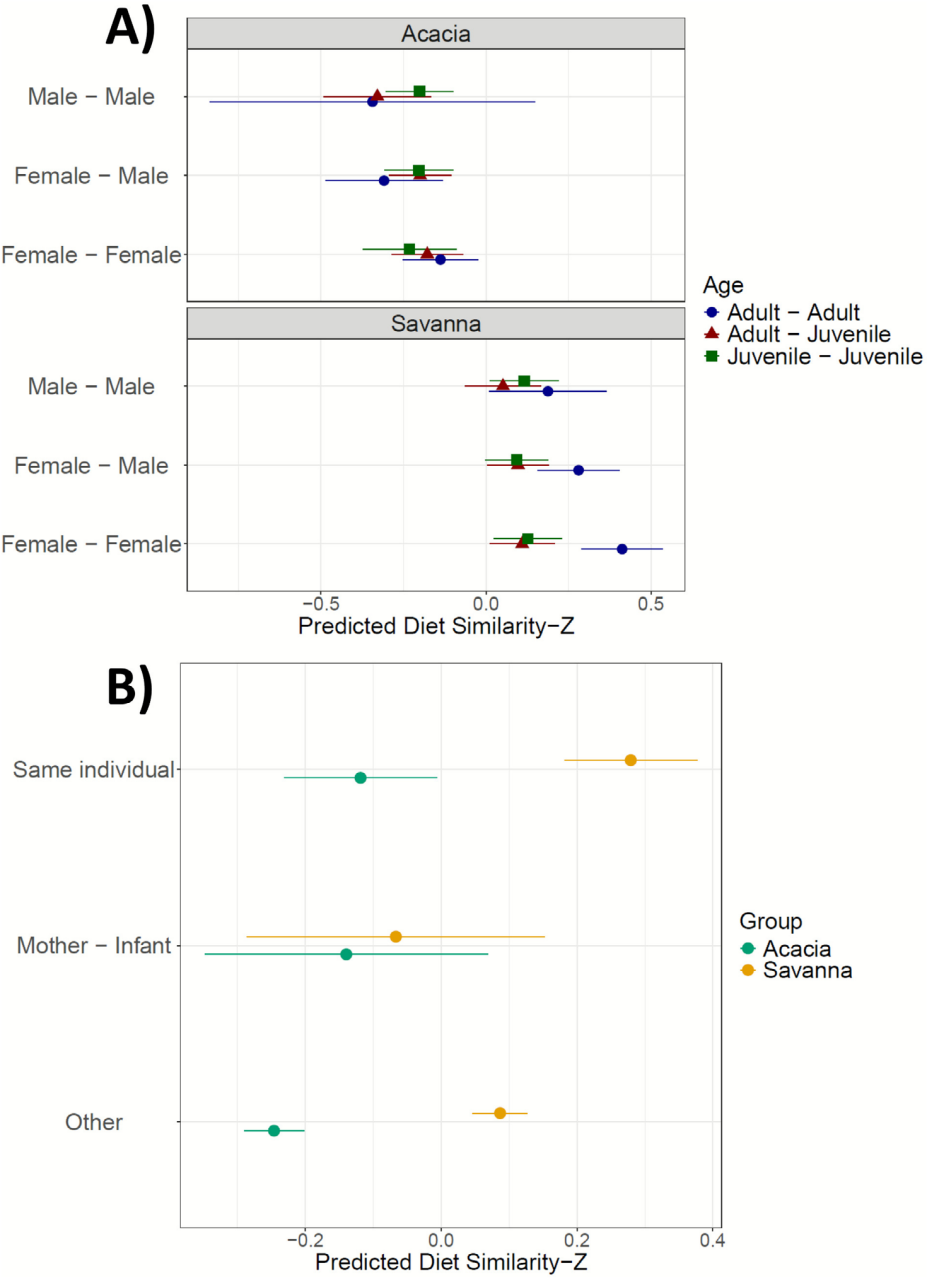
Predicted diet of standardised similarity model for (A) interactions between groups, sex and age on standardised similarity with lines corresponding to a sex combination and shape colour to an age combination, and for (B) interaction between groups and relationship on standardised similarity, with lines corresponding to a relationship combination for each group.

We then ran two models as previously described. For Model (1), including the most variables, we observed a significant difference between groups in terms of sample similarity (Chi‐sq = 319.15, df = 1, *p‐value* < 2.2e‐16, Figure 9A), indicating that individuals in the Savanna group had more similar diets compared to those in the Acacia group. Additionally, the interaction between ages was significant (Chi‐sq = 23.97, df = 2, *p‐value* = 6.23e‐06), as was the interaction between age and group (Chi‐sq = 18.89, df = 2, *p‐value* = 7.906e‐05), and the interaction between age and sex (Chi‐sq = 3.79, df = 4, *p‐value* = 0.044). This suggests that the age of individuals within a group influenced dietary similarity, and that sex also influenced similarity in relation to age, with adult females in the Savanna group exhibiting more similar diets than other group members.

For Model (2), we again found that similarity between individuals was strongly dependent on the group (Model (2)—Group; Chi‐sq = 289.38, df = 1, *p‐value* < 2.2e‐16, Figure 9B) as well as on the relationship between faecal samples (Model (2)—Relationship; Chi‐sq = 19.74, df = 2, *p‐value* = 5.179e‐05). As shown in Figure 9B, faecal samples from the same individual indeed had higher similarity compared to faecal samples from other individuals. However, we found a significant overlap in diet between the two study groups when comparing faecal samples from mothers and their infants. The similarity for this category was higher than that among Acacia individuals but lower than that among Savanna individuals.

## 4 Discussion

Vervet monkeys demonstrate broad behavioural flexibility enabling them to thrive in urban environments (Barnes et al. 2025; Johnson‐Ulrich and Forss 2025). However, the extent to which their diet changes with anthropisation remains largely unknown. In this study, we compared two methods (metabarcoding of eDNA and observational data) to measure the proportion of human food of two monkey groups and analysed their different dietary patterns between and within them. The two methods showed consistent results, and our data enabled the identification of differences among groups and individuals, potentially influenced by the presence of anthropogenic food sources within their habitat.

### 4.1 Observational and Faecal Data Comparisons

Our results from faecal data of semi‐urban vervet monkeys proved reliable and consistent with the observational data, aligning with findings reported by Brun et al. (2022) on the diet of a wild population of vervet monkeys (Figure 6). However, although observational data allowed for a clear distinction between natural versus non‐natural dietary resources and a high level of taxonomic resolution, they remained limited in detecting species diversity (Figure 5). These limitations are likely due to the broad diet of the vervet monkeys (Chapman et al. 2016), consisting of many different plant species (some of which are rare and likely new even to trained observers) as well as a variety of vertebrates (Figure 3). Identifying all consumed species is therefore challenging and time‐intensive. Visual identification is further complicated by dietary elements like seeds or sap, which can be difficult to distinguish visually. Additionally, brief observation events can overlook species such as grasses or small flowering plants, which in our study potentially explains the absence of taxa like *Apiales*, *Oxalidales*, and *Poales* in the observational data compared to the eDNA patterns (Figure 4; Figure 5). Furthermore, urban barriers and constructions, such as houses, as well as ecological conditions like marshes or dense terrain, can obstruct the path and thereby challenge the continuous tracking of monkeys' food intake. It is plausible that some dietary species in our study were only present in areas inaccessible to researchers, limiting detection in the observational dataset. Moreover, we considered time as a proxy for relative abundance; however, the consumption of fibrous and hard‐to‐open plant species, such as *Strelitzia* sp., requires considerable handling time by the monkeys, which potentially contributed to an overestimation of their dietary importance in the observational data (Figure 4).

The results from faecal samples provided a broader coverage of ingested species compared to observational data, which often identified only a few species dominating the diet (Figure 5). Nonetheless, the species detected in each dataset yielded similar proportional results (Figure 6). The DNA metabarcoding method also enabled the identification of some vertebrate species, though their consumption was found to be relatively rare (Figure 3 and Figure S2). However, faecal data offered less precise taxonomic resolution overall, particularly for certain plant families like the *Asterales* group (Figure 5). Also, *Harpephyllum caffrum* and *Protorhus longifolia*, found only in the observational data, were likely grouped within the Anacardiaceae family, and part of the data for *Vachellia* sp. may have been included in the Fabaceae family (Figure S2). This lack of precision may arise from low interspecific variability in the metabarcodes, as the *Sper01* primer target region is not consistently discriminatory; consequently, species with shorter sequences or close phylogenetic relationships may be indistinguishable at the species or genus level (Schneider et al. 2021; Taberlet et al. 2018). The reduced resolution for certain taxa complicated the interpretation of human food consumption, making the determination of the proportion of anthropogenic food in the vervet monkeys' diet somewhat subjective, with the handling of DNA metabarcoding data significantly affecting the results (Calderón‐Sanou et al. 2020; Figure 7 and Figure S2).

Observational data indicated that 4.5% of the diet consisted of human food over the 4 months of data collection, which was almost double the 2.3% found in the faecal samples (Figure 7). However, it is highly likely that a portion of the 12.2% of food of unknown origin corresponds to a certain extent to anthropogenic food. Consequently, the 2.3% estimate represents a minimum value that could increase to up to 8% if the unidentified portion were proportionally allocated between the two categories. Vervet monkeys frequently stop when encountering trash bins and often spend considerable time foraging through them. Thus, the results obtained appear coherent, considering that non‐natural resources can contribute up to one‐third of vervet monkeys' daily caloric intake (Patterson et al. 2019). Yet, some items may not have been detected in the faecal samples because of the degradation of DNA from highly processed foods such as cooked potatoes (Schneider et al. 2021), potentially underestimating the proportion of human‐derived DNA (e.g., dairy products, fried or cooked foods). On the other hand, detecting human food consumption through observations can be challenging when consumed indoors or in trash bins, where monkeys can be out of sight. Tracking them also becomes harder around properties where human food is typically found, as they often move out of the observers' view, leading to limited data collection. Therefore, the choice of methods depends on the specific circumstances and research objectives. Observational data provide higher certainty to distinguish between anthropogenic and natural food sources, whereas faecal data offer an overview over 24 h with a broader coverage among vervet monkeys.

### 4.2 Molecular Methodological Limitations

Molecular metabarcoding offers great potential for estimating biomass and abundance in ecological studies, but remains subject to several biases. Digestive processes, for instance, can distort the correlation between ingested biomass and read counts (Deagle et al. 2005, 2013; Clare 2014). This could be the reason behind the dominance of *Ficus* sp. in our results (Figure 4 and Figure S2), as they produce large dense fruits that may contain a high amount of DNA or that might simply be more resistant to degradation during digestion. Also, our study focused on plants and vertebrates, excluding fungi, lichens, and invertebrates (Figure S4), which are known dietary items of vervet monkeys (Tournier et al. 2014). Furthermore, some species detected through observations were not identified molecularly, potentially highlighting issues with incomplete databases (Furlan et al. 2020; Taberlet et al. 2012). The low number of reads obtained for vertebrates is likely attributable to primer *Vert01* amplifying a high proportion of DNA from humans or vervet monkeys, despite the use of a blocking oligonucleotide. Thus, observational methods could offer more reliable abundance data (Brun et al. 2022) and provide detailed information on consumed items, such as specific plant parts (e.g., fruit, flowers, and leaves) and vertebrates (e.g., meat, dairy products, and eggs; Pompanon et al. 2012; Rees et al. 2014; Schneider et al. 2021; Figure S5). Therefore, both methods could be beneficial for analysing diet and abundance in conservation applications, depending on the required level of precision, coverage, and the taxa targeted.

### 4.3 Dietary Intergroup Comparisons

We observed that the Savanna group had a higher proportion of human food consumption compared to the Acacia group (Figure 8A), suggesting a potential difference in dietary patterns between the two groups (observed through the NMDS analysis; Figure 8B). However, this observation should be interpreted with caution, as it may be influenced by a sampling bias between the two groups (Tables S2 and S3). Moreover, although the groups' territories overlap to some extent (Figure 2), it is possible that the observed group difference can be explained by an unequal distribution of plant species between the territories (Schneider et al. 2023). The natural plants in the Savanna territory might have lower nutritional values, leading individuals to consume more human food items to compensate. Moreover, although the dietary difference between these two groups might be explained by the consumption of human food, it could also be due to the Acacia group having a significant portion of their territory outside the estate, which could reduce the opportunities to enter houses or exploit trash bins, increasing the consumption of natural food present in the outside forest area. Few studies have examined the variation in foraging behaviour between neighbouring primate groups (Quéméré et al. 2013; Samuni et al. 2020; Tournier et al. 2014). One study noted that intragroup variations were correlated with group size and were reduced in cases where the number of individuals was smaller (Schneider et al. 2023). Given the very small group sizes in our study, this supports the idea that the diets of the two monkey groups potentially present significant differences, with intergroup variation likely being higher than intragroup variation.

Previous work on this population has shown that vervet monkeys in a semi‐urban environment spend a significant amount of time seeking anthropogenic food (Thatcher et al. 2020) and have a reduced need to spatially explore their environment to satisfy their caloric requirements (Patterson et al. 2019). Individuals in the Savanna group exhibited greater dietary similarity compared to those in the Acacia group (Figure 9A). One hypothesis is that the Savanna group, which in our dataset consumed more human food, benefits more from the high energy content of anthropogenic resources (Hoffman and O'Riain 2012; Patterson et al. 2018; Saj et al. 2001). This could reduce the need for extensive foraging, leading them to rely on easily accessible species, thereby decreasing dietary diversity and increasing similarity. Conversely, the Acacia individuals, consuming less human food, would need to compensate for their caloric intake with more natural food. This would lead them to spatially explore their territory more extensively, consuming plant species more opportunistically, thereby increasing the diversity of consumed species and reducing dietary similarity among individuals. Existing research by Sengupta and Radhakrishna (2018) highlighted that access to anthropogenic food impacts habitat use in rhesus macaques ( *Macaca mulatta* ), with individuals spending more time in human‐modified areas during periods of food provisioning. Similarly, chacma baboons (*Papio hamadryas ursinus*) showed an increase in the time spent foraging in their natural environment when deprived of anthropogenic resources (Mazué et al. 2023). However, precise assessments of caloric intake and nutritional values of the different dietary items would be necessary to confirm this hypothesis. Hence, human food consumption can lead to vervet monkeys exploiting natural resources less, a phenomenon already demonstrated by previous research (Jaman and Huffman 2013; Saj et al. 1999). Nonetheless, it is still possible that the explanation lies in the presence of more nutritious and abundant natural plant species in the Savanna territory compared to the Acacia territory.

### 4.4 Dietary Intragroup Comparisons

Our hypotheses were based on the idea that an individual does not consume resources randomly but follows consistent dietary habits, and that infants learn socially what to eat from their mothers, resulting in greater dietary similarity between mother‐infant pairs (van de Waal et al. 2012, 2013, 2014). We found that, for both groups, faecal samples from the same individual were more similar to each other than to those from other group members, supporting our hypothesis (Figure 9B).

In the Acacia group, the diet of mother‐infant pairs was more similar to each other than to the rest of the group. In contrast, in the Savanna group, the diet of mother‐infant pairs was less similar to each other compared to the rest of the group but showed a similar level of similarity to the Acacia mother‐infant pairs. Despite the large confidence intervals, which can be explained by differences in sample sizes among the categories (Figure S3), one explanation of our observations is that mothers and their infants might be more cautious in their foraging behaviour. Consequently, mothers and infants may avoid potential risks involved in exploiting human food sources and thereby foraging more extensively for natural resources (Thatcher et al. 2020), leading the mother‐infant pairs in the Savanna group to have a diet more similar to that of the Acacia group.

This may suggest that the territories of Acacia and Savanna are not significantly different in terms of species distribution and further confirms that the dietary differences between the groups are more closely related to a potentially higher consumption of anthropogenic resources by the Savanna group, as also indicated by the substantial overlap in their territories and the results shown in Figure 8. However, the potential caution exhibited by mothers and their infants could also be a matter of individual monkey personality, hierarchy, preference for natural food, or different energy needs because of the reproductive state of the females (Schneider et al. 2023).

These findings highlight the reliance of urbanised primate populations on anthropogenic resources. Consequently, measures aimed at improving waste management and discouraging intentional provisioning are necessary to reduce access to human food sources. Implementing these measures, together with maintaining natural foraging patches, may help mitigate human–wildlife conflicts, decrease dependence on anthropogenic food, and prevent the potential health risks for primates ingesting highly processed food (see Mazué et al. 2023; Moy et al. 2023; Sengupta and Radhakrishna 2018).

## 5 Conclusion

Metagenomic data on monkey diets have proven useful for addressing behavioural questions related to inter‐ and intra‐group variability and could also support the study of social learning in a non‐experimental context. Our study further highlights the importance of reliable dietary data, which are crucial for conservation strategies (Aleixo‐Pais et al. 2023; Henger et al. 2022; Mas‐Carrió et al. 2024; Monterroso et al. 2019; Schwarz et al. 2018), especially in a world of accelerating urbanisation that increasingly affects the distribution of natural resources. The application of DNA metabarcoding in conservation programs could therefore be a valuable addition to our understanding of resource distribution. Our study demonstrates the advantages of combining two complementary methods to characterise the complex diet of a generalist species and, to our knowledge, represents the first application of an environmental DNA approach to define dietary habits in a generalist species within an urbanised ecosystem. Given that anthropogenic food exploitation is a key factor in increasing interactions between humans and wildlife (Thatcher et al. 2020), more specific studies are needed to elucidate the extent of the impact of human food consumption on inter‐group dietary variation and its influence on the exploitation of natural resources.

## Author Contributions

**Joey Felsch:** conceptualization (equal), data curation (supporting), formal analysis (equal), investigation (equal), methodology (equal), visualization (lead), writing – original draft (lead), writing – review and editing (equal). **Eduard Mas‐Carrió:** conceptualization (equal), formal analysis (equal), methodology (equal), software (lead), supervision (lead), writing – review and editing (equal). **Stéphanie Mercier:** data curation (lead), investigation (equal), project administration (lead), writing – review and editing (equal). **Judith Schneider:** writing – review and editing (equal). **Sofia Forss:** conceptualization (equal), funding acquisition (equal), resources (equal), writing – review and editing (equal). **Erica Van de Waal:** conceptualization (equal), funding acquisition (equal), resources (equal), writing – review and editing (equal). **Luca Fumagalli:** conceptualization (equal), funding acquisition (equal), resources (equal), writing – review and editing (equal).

## Funding

This work was supported by Kone Foundation, 202006900. Förderung des Akademischen Nachwuchses, Gebauer Stiftung. Research Council under the European Union's Horizon 2020 research and innovation programme for the ERC ‘KNOWLEDGE MOVES’ starting grant 949379. Schweizerischer Nationalfonds zur Förderung der Wissenschaftlichen Forschung, 310030_192512, CRSK‐3 220769, PZ00P3 202052.

## Conflicts of Interest

The authors declare no conflicts of interest.

## Supporting information


**Appendix S1:** Supporting Information.

## Data Availability

The DNA metabarcoding and observational data, as well as the results of the local plants sequencing, are available on DRYAD (https://doi.org/10.5061/dryad.15dv41p8p).
