# Supplementary material for: Investigating Anthropogenic and Social Influences on Diet of Semi‐Urban Vervet Monkeys Using DNA Metabarcoding
Source: Ecol Evol. 2026 Feb 3;16(2):e73008. doi: 10.1002/ece3.73008 (PMC12868386; doi:10.1002/ece3.73008)
Supplement: Supplementary file 1 — Appendix S1: Supporting Information. [file ECE3-16-e73008-s001.docx]

**Supplementary**

| **Group** | **Age** | **Sex** | **Monkey name** | **Samples** |
| --- | --- | --- | --- | --- |
| **Savanna** | Adult | Male | *Whisky* | 11 |
|  |  |  | *Jägermeister* | 9 |
|  |  |  | *Gin* | 13 |
|  |  | Female | *Margerita**** | 4 |
|  |  |  | *Ruby* | 12 |
|  |  |  | *Piña Colada**** | 1 |
|  |  |  | *Caipiriña* | 11 |
|  |  |  | *Black Russian**** | 15 |
|  |  |  | *Tequila**** | 13 |
|  | 3 years old | Male | *WhiteDot* | 16 |
|  |  | Female | *Sangria* | 20 |
|  | 2 years old | Male | *CuriousBoy* | 7 |
|  |  |  | *DentedChin* | 10 |
|  |  | Female | *GoateeGirl* | 14 |
|  |  |  | *CutePinkyGirl* | 11 |
|  | 1 year old | Male | *Elf* | 17 |
|  |  |  | *WhitePatches* | 10 |
|  |  | Female | *SpottedChin* | 13 |
|  | Infant | Male | *Mojito* | 15 |
|  |  |  | *Apérol* | 7 |
|  |  | Female | *Tornado* | 14 |
|  |  |  | *Paloma* | 11 |
|  |  |  | *Banshee* | 8 |
| **Acacia** | Adult | Male | *Xyris* | 8 |
|  |  | Female | *Marula**** | 9 |
|  |  |  | *Vachellia* | 9 |
|  |  |  | *Agave* | 12 |
|  |  |  | *Dahlia**** | 15 |
|  |  |  | *Cherry**** | 10 |
|  |  |  | *Kauri**** | 9 |
|  | 4 years old | Male | *Ziziphus* | 10 |
|  | 3 years old | Male | *Quince* | 8 |
|  |  | Female | *Rosa* | 11 |
|  | 2 years old | Male | *Fig* | 8 |
|  |  | Female | *Gumy* | 2 |
|  | 1 year old | Male | *Jalapeño* | 4 |
|  |  |  | *Yucca* | 17 |
|  |  | Female | *Angelica* | 8 |
|  |  |  | *Palmera* | 11 |
|  | Infant | Male | *Cactus* | 12 |
|  |  |  | *Kiwi* | 6 |
|  |  |  | *Dragon* | 11 |
|  |  | Female | *Macadamia* | 5 |
|  |  |  |  |  |

**Table S1:** Details of group composition for individuals who contributed to sample collection, with the corresponding number of faecal samples for each individual. The *** means that the individual has a baby with a name beginning with the same letter as her.

| **Period** | **Aug.15^th^-Sep.16^th^** | | **Sep.17^th^-Oct.18^th^** | | **Oct.19^th^-Nov.20^th^** | | **Nov.21^th^-Dec.22^th^** | | **Total** | |
| --- | --- | --- | --- | --- | --- | --- | --- | --- | --- | --- |
| **Data** | *Faecal* | *Obs.* | *Faecal* | *Obs.* | *Faecal* | *Obs.* | *Faecal* | *Obs.* | *Faecal* | *Obs.* |
| **Acacia** | 44 | 37/7 | 40 | 103/36 | 35 | 71/11 | 66 | 94/18 | **185** | **305/72** |
| **Savanna** | 87 | 81/21 | 52 | 86/21 | 65 | 101/22 | 58 | 67/10 | **262** | **335/74** |

**Table S2:** Number of occurrences of faecal and observational data over the four months of data collection for both groups. For the observational data, the first number represents the total number of focal samples conducted, and the second number indicates the number of aborted samples (less than 15 minutes). The shortest focal sample lasted 67 seconds.

| **Groups** | **Adult males** | | **Adult females** | | **Juveniles** | | **Total** | |
| --- | --- | --- | --- | --- | --- | --- | --- | --- |
|  | *Monkeys* | *Samples* | *Monkeys* | *Samples* | *Monkeys* | *Samples* | *Monkeys* | *Samples* |
| **Acacia** | 1 | 8 | 6 | 64 | 13 | 113 | **20** | **185** |
| **Savanna** | 3 | 33 | 6 | 56 | 14 | 173 | **23** | **262** |

**Table S3:** Individuals who contributed to the faecal sample collection with the number of faecal samples corresponding to each class of monkey.

| **Reads** | **Order** | **Family** | **Species** |
| --- | --- | --- | --- |
| 0 | Apiales | Araliaceae | ***Cussonia Sphaerocephala, Cussonia zuluensis*** |
| *NA*  *NA*  *NA*  0  *NA*  *NA*  *NA*  *NA* | Asparagales  Asparagales  Asparagales  Asparagales  Asparagales  Asparagales  Asparagales  Asparagales | Asphodelaceae  Asphodelaceae  Asphodelaceae  Asphodelaceae  Asphodelaceae  Asphodelaceae  Asphodelaceae  Asphodelaceae | *Aloe chabaudii****  *Aloe cooperi****  *Aloe ferox****  *Aloe marlothii****  *Aloe pluridens****  *Aloe thraskii****  *Aloe vanbalenii****  *Aloiampelos tenuior**** |
| 0  0  0 | Caryophyllales  Ericales  Ericales | Aizoaceae  Sapotaceae  Sapotaceae | *Carpobrotus dimidiatus****  ***Mimusops caffra, Mimusops obovata***  *Sideroxylon inerme* |
| 62,792  *NA* | Fabales  Lamiales | Fabaceae  Bignoniaceae | *Albizia adianthifolia*  *Tecomaria capensis* |
| 0  0  0  0 | Lamiales  Lamiales  Lamiales  Malpighiales | Boraginaceae  Lamiaceae  Oleaceae  Achariaceae | *Cordia caffra*  *Leonotis leonurus*  *Jasminum multipartitum*  *Xylotheca kraussiana* |
| 0  *NA* | Malpighiales  Malpighiales | Clusiaceae  Clusiaceae | *Garcinia gerrardii*  *Garcinia livingstonei* |
| 0  0  0 | Malpighiales  Malpighiales  Malpighiales | Euphorbiaceae  Phyllanthaceae  Salicaceae | *Macaranga capensis*  *Antidesma venosum*  *Dovyalis longispina* |
| 0 | Malvales | Malvaceae | *Dombeya rotundifolia* |
| 0  0  819 | Malvales  Myrtales  Myrtales | Malvaceae  Myrtaceae  Myrtaceae | *Hibiscus pedunculatus*  *Eugenia capensis*  ***Syzygium cordatum, Syzygium gerrardii*** |
| 1,148,237 | Rosales | Moraceae | ***Ficus burkei, Ficus trichopoda*** |
| 0 | Rosales | Moraceae | ***Ficus burtt-davyi, Ficus lutea, Ficus polita*** |
| 0 | Sapindales | Anacardiaceae | *Searsia gueinzii* |
| 0 | Sapindales | Rutaceae | *Teclea gerrardii* |

**Table S4:** Species collected for the local database. The *** indicates that plant samples were preserved in 95% ethanol rather than dried with silica gel beads. In bold are the species with identical sequences and the lines with an *NA* correspond to species that did not produce exploitable sequences.


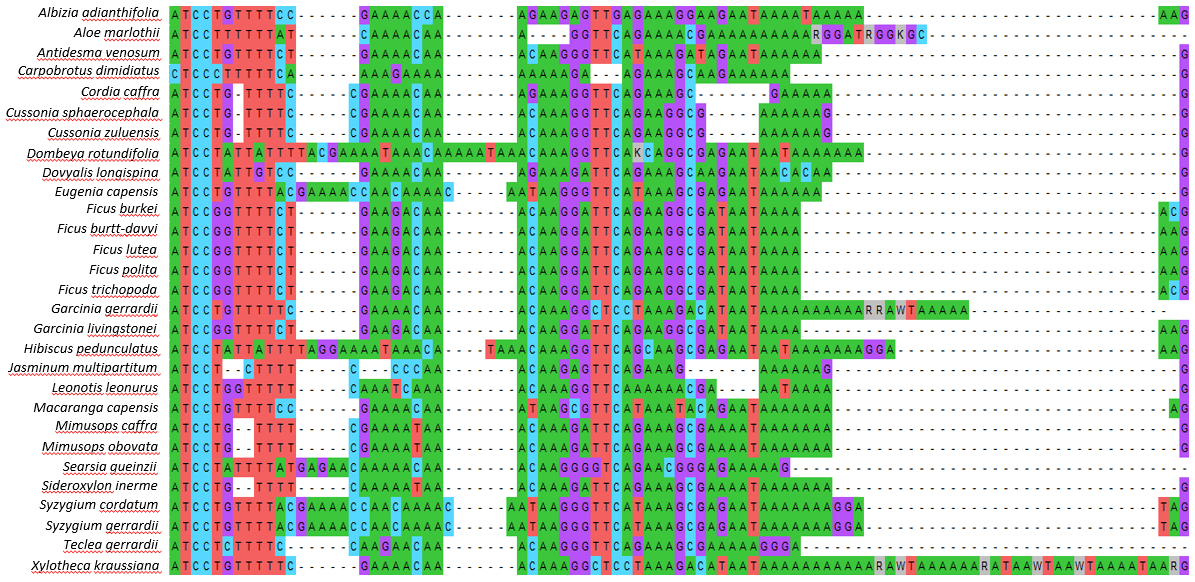
**Figure S1:** Alignment of the sequences obtained from the DNA of collected plant samples for the local database.

| **Metabarcode** | **Species** |
| --- | --- |
| *Sper01* | *Abies alba*, *Acer campestre*, *Briza media*, *Capsella bursa-pastoris*, *Carpinus betulus*, *Fraxinus excelsior*, *Geranium robertianum*, *Lonicera xylosteum*, *Lotus corniculatus*, *Picea abies*, *Populus tremula*, *Rhododendron ferrugineum*, *Rosa canina*, *Rumex acetosa*, *Salvia pratensis*, *Taxus baccata* |
| *Vert01* | A*mphiprion ocellaris*, *Cynoliseus patagonus*, *Myotis capaccini*, *Rana kurkensis*, *Rana sphenocephalis* |
| *Vert01* | *Macropus dorsalis*, *Macropus gigantus*, *Macropus rufogrisen*, *Myotis capaccini*, *Myotis myotis* |

**Table S5:** Species included in positive controls for Sper01 and Vert01 assays.

| **Primer** | **Score** | | **Order** | **Family** | **Subfamily level** | **Sum of RRA** |
| --- | --- | --- | --- | --- | --- | --- |
| ***Sper01***  ***Vert01*** | | **0**  **0.5**  **1**  **0**  **1** | **Apiales**  **Arales**  **Asparagales**  **Asterales**  **Canellales**  **Caryophyllales**  **Commelinales**  **Ericales**  **Fabales**  **Fagales**  **Gentiales**  **Lamiales**  **Liliales**  **Malpighiales**  **Malvales**  **Myrtales**  **Oxalidales**  **Pandanales**  **Pinales**  **Poales**  **Rosales**  **Sapindales**  **Solanales**  **Vitales**  **Zingiberales**  **Apiales**  **Arecales**  **Asparagales**  **Asterales**  **Caryophyllales**  **Cucurbitales**  **Ericales**  **Fabales**  **Gentianales**  **Laurales**  **Malpighiales**  **Poales**  **Rosales**  **Rosidae (subclass)**  **Sapindales**  **Solanales**  **Vitales**  **Zingiberales**  **Asparagales**  **Brassicales**  **Ericales**  **Fabales**  **Fagales**  **Lamiales**  **Malpighiales**  **Myrtales**  **Poales**  **Rosales**  **Sapindales**  **Solanales**  **Violales**  **Zingiberales**  **Artiodactyla**  **Columbiformes**  **Passeriformes**  **Squamata**  **Artiodactyla**  **Galliformes** | Apiaceae  Araliaceae  Araceae  Asparagaceae  Asteraceae  Cannellaceae  Plumbaginaceae  Polygonaceae  Commelinaceae  Actinidiaceae  Ebenaceae  Lecythidaceae  Sapotaceae  Fabaceae  Fagaceae  Juglandaceae  Apocynaceae  Rubiaceae  Acanthaceae  Bignoniaceae  Oleaceae  Stilbaceae  Verbenaceae  Smilacaceae  Euphorbiaceae  Salicaceae  Malvaceae  Myrtaceae  Onagraceae  Oxalidaceae  Pandanaceae  Pinaceae  Poaceae  Typhaceae  Cannabaceae  Moraceae  Urticaceae  Anacardiaceae  Meliaceae  Sapindaceae  Convolvulaceae  Vitaceae  Strelitziaceae  Apiaceae  Arecaceae  Aspargaceae  Asteraceae  Polygonaceae  Cucurbitaceae  Theaceae  Fabaceae  Rubiaceae  Lauraceae  Linaceae  Passifloraceae  Poaceae  Moraceae  Rosaceae  Rutaceae  Sapindaceae  Solanaceae  Vitaceae  Amaryllidaceae  Brassicaceae  Ericaceae  Fabaceae  Betulaceae  Pedaliaceae  Linaceae  Myrtaceae  Bromeliaceae  Poaceae  Rosaceae  Anacardiaceae  Solanaceae  Caricaceae  Musaceae  Bovidae  Columbidae  Estrildidae  Nectariniidae  Ploceideae  Zosteropidae  Gekkonidae  Bovidae  Phasianidae | *Centella asiatica*  *Hydrocotyle sp.*  Agavoideae  Asteroideae  *Plumbago sp.*  *Rumex sp.*  *Callisia sp.*  *Commelina benghalensis*  *Actinidia sp.*  *Diospyros sp.*  Acacieae  *Albizia adianthifolia*  Caesalpinioideae  Desmodieae  *Desmodium gangeticum*  *Desmodium incanum*  *Dichrostachys cinerea*  *Eriosema sp.*  *Erythrina sp.*  *Leucaena sp.*  *Senna sp.*  *Vachellia sp.*  *Quercus sp.*  Carrisseae  *Cynanchum sp.*  *Voacanga sp.*  *Tabernaemontana sp.*  *Canthium sp.*  *Asystasia sp.*  *Tecomaria capensis*  *Jasminum sp.*  *Noronhia peglerae*  *Halleria lucida*  *Halleria sp.*  *Smilax sp.*  *Mercurialis annua*  *Tragia urticifolia*  *Dovyalis sp.*  *Syzygium sp.*  *Oenothera sp.*  *Oxalis sp.*  *Piceae sp.*  *Pinus sp.*  *Bromus sp.*  *Campeiostachys nutans*  *Cenchrus sp.*  *Cynodon sp.*  *Eleusine multiflora*  *Garnotia sp.*  *Leersia sp.*  *Paspalum notatum*  *Phragmites austalis*  *Poa sp.*  *Trichoneura lindleyana*  *Typha sp.*  *Celtis sp.*  *Ficus sp.*  *Morus sp.*  *Didymodoxa caffra*  Anacardioideae  *Protorhus longifolia*  *Schinus terebinthifolia*  *Ekebergia capensis*  *Melia azedarach*  *Deinbollia oblongifolia*  *Hewittia sp.*  *Ipomoea cairica*  *Cissus sp.*  *Ravenala madagascariensis*  *Strelitzia sp.*  Apioideae  *Asparagus sp.*  *Lactuca sp.*  Polygonoideae  *Camellia sp.*  Faboideae  Phaseoleae  *Linum sp.*  *Passiflora sp.*  *Digitaria sp.*  *Eleusine sp.*  Paniceae  *Citrus sp.*  *Murraya sp.*  *Allium sp.*  *Brassica oleraceae*  Vaccinieae  *Arachis sp.*  *Glycine max*  *Lens culinaris*  *Vigna unguiculata*  *Sesamum sp.*  *Linum usitatissimum*  *Psidium guajava*  *Ananas comosus*  *Avena sp.*  *Hordeum vulgare*  *Oryza sativa*  *Panicum miliaceum*  *Sorghum sp.*  *Prunus sp.*  *Pyrus sp.*  *Mangifera indica*  *Capsicum sp.*  *Solanum lycopersicum*  *Carica papaya*  *Musa balbisiana*  *Musa sp.*  *Sylvicapra grimmia*  *Columba livia*  *Lonchura cucullata*  *Nectarinia venusta*  *Ploceus bicolor*  *Ploceus sp.*  *Hemidactylus mabouia*  *Bos taurus*  *Ovis aries*  *Gallus gallus* | 1.1  0.052  0.0064  0.026  0.11  9.25  0.0063  0.017  0.071  0.26  0.021  0.021  0.049  0.019  0.095  2.38  0.0095  3.59  0.011  0.028  0.0053  1.74  0.065  0.0039  76.21  0.12  0.0055  0.2  0.00071  0.016  3.47  0.32  0.25  0.18  0.39  0.39  0.83  4.44  0.6  0.004  0.00066  0.0024  11.38  0.82  0.47  0.77  0.0015  0.0097  0.55  0.0076  0.062  0.007  0.66  0.0011  8.23  0.34  0.008  0.013  0.13  0.39  0.039  0.009  0.0022  0.033  0.034  0.028  0.67  0.11  0.0066  2.05  0.0042  7.23  130.88  2.99  0.058  2.21  11.81  0.018  3.56  0.014  1.94  24.88  0.44  1.52  1.16  0.0021  0.18  0.0024  59.7  1.52  0.0022  0.03  0.1  0.096  0.077  0.00085  0.41  0.0046  33.77  0.82  0.004  0.013  0.87  0.032  0.1  6.44  0.011  01.56  2.17  0.0052  0.32  0.17  0.0012  0.39  0.013  0.064  0.57  4.81  0.019  0.00074  0.66  0.0023  0.045  0.048  0.0042  0.48  0.0055  0.0059  0.015  0.0014  0.16  0.019  0.0088  0.029  0.0018  0.73  0.28  0.16  0.18  0.083  0.28  0.81  5.15  0.014  0.00059  0.025  0.019  0.12  0.017  0.44  0.0046  0.034  0.0052  0.043  0.0029  0.89 |

**Table S6:** Sum of relative read abundance for each taxon discovered based on the scoring system and primer used. A score of 0 represented a natural resource, a score of 0.5 represented a resource that could be either natural or anthropogenic, and a score of 1 represented an anthropogenic resource. *Sper01* primer targeted plant taxa, whereas *Vert01* targeted vertebrate taxa.


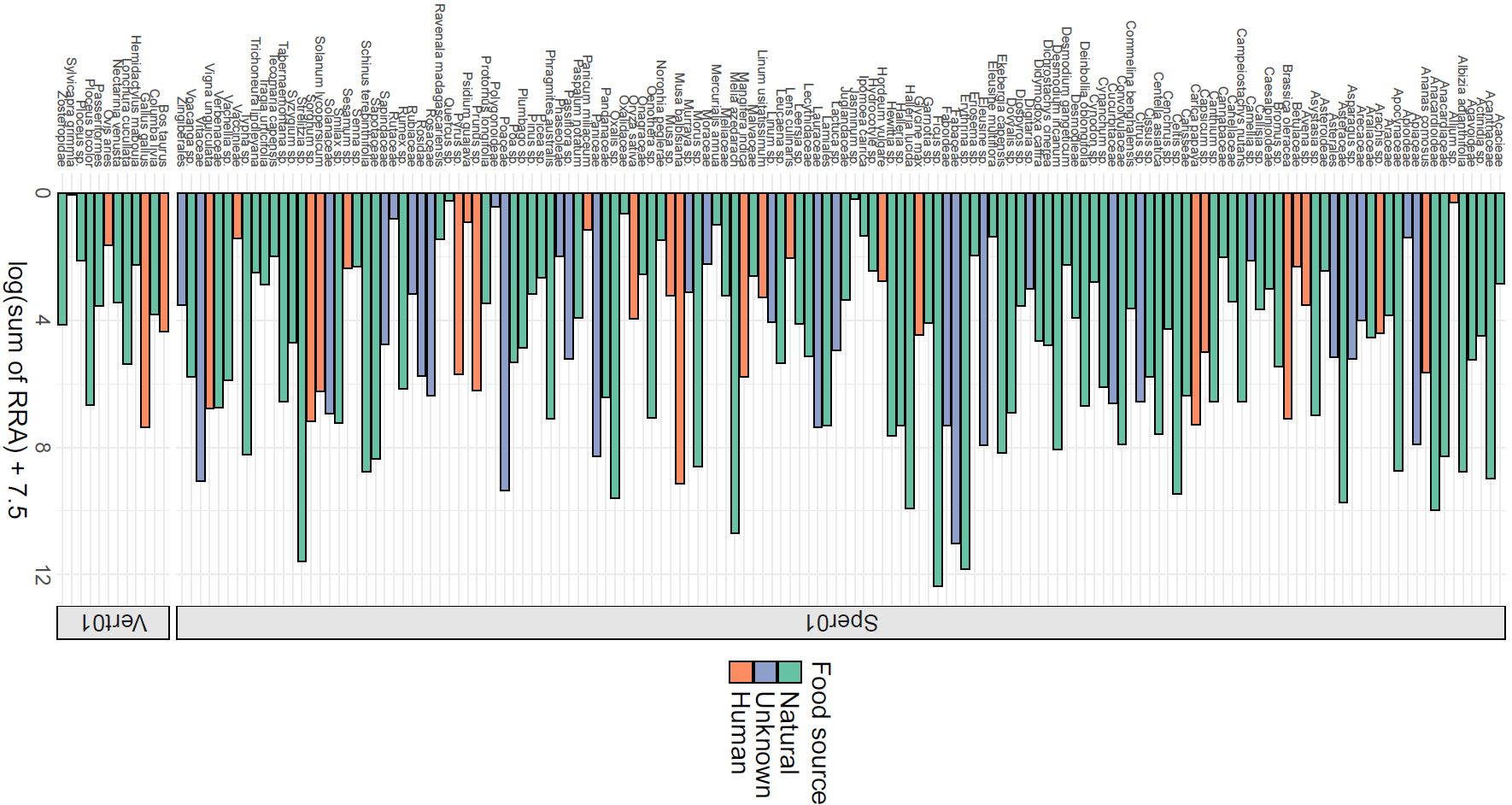
**Figure S2:** Logarithm of the sum of relative read abundance plus 7.5 for each taxon for *Sper01* and *Vert01* found in faecal samples represented in green for the natural resources, in orange for the human resources and in blue for the unknown origin resources.

|  | **Species** | **Genus** | **Tribes** | **Subfamily** | **Families** | **Order** | **Subclass** |
| --- | --- | --- | --- | --- | --- | --- | --- |
| *Sper01* resolution | 38 | 57 | 6 | 7 | 32 | 3 | 1 |
| Plant diversity | 38 | 90 | NA | NA | 60 | 31 | NA |
| *Vert01* resolution | 9 | 1 | NA | NA | 1 | 1 | NA |
| Vertebrate diversity  Obs. plant resolution  Obs. plant diversity | 9  28  28 | 9  4  24 | NA  NA  NA | NA  NA  NA | 8  NA  16 | 5  NA  11 | NA  NA  NA |

**Table S7:** Number of different taxa for *Sper01*, *Vert01*, and observational data resolution and diversity at each taxonomic level for plants and vertebrates in faecal data, and for plants only in observational data. Resolution is the exact number of taxa per taxonomic rank found, while diversity is the sum of all taxa found of taxonomic ranks equal to or lower than a given taxonomic rank.


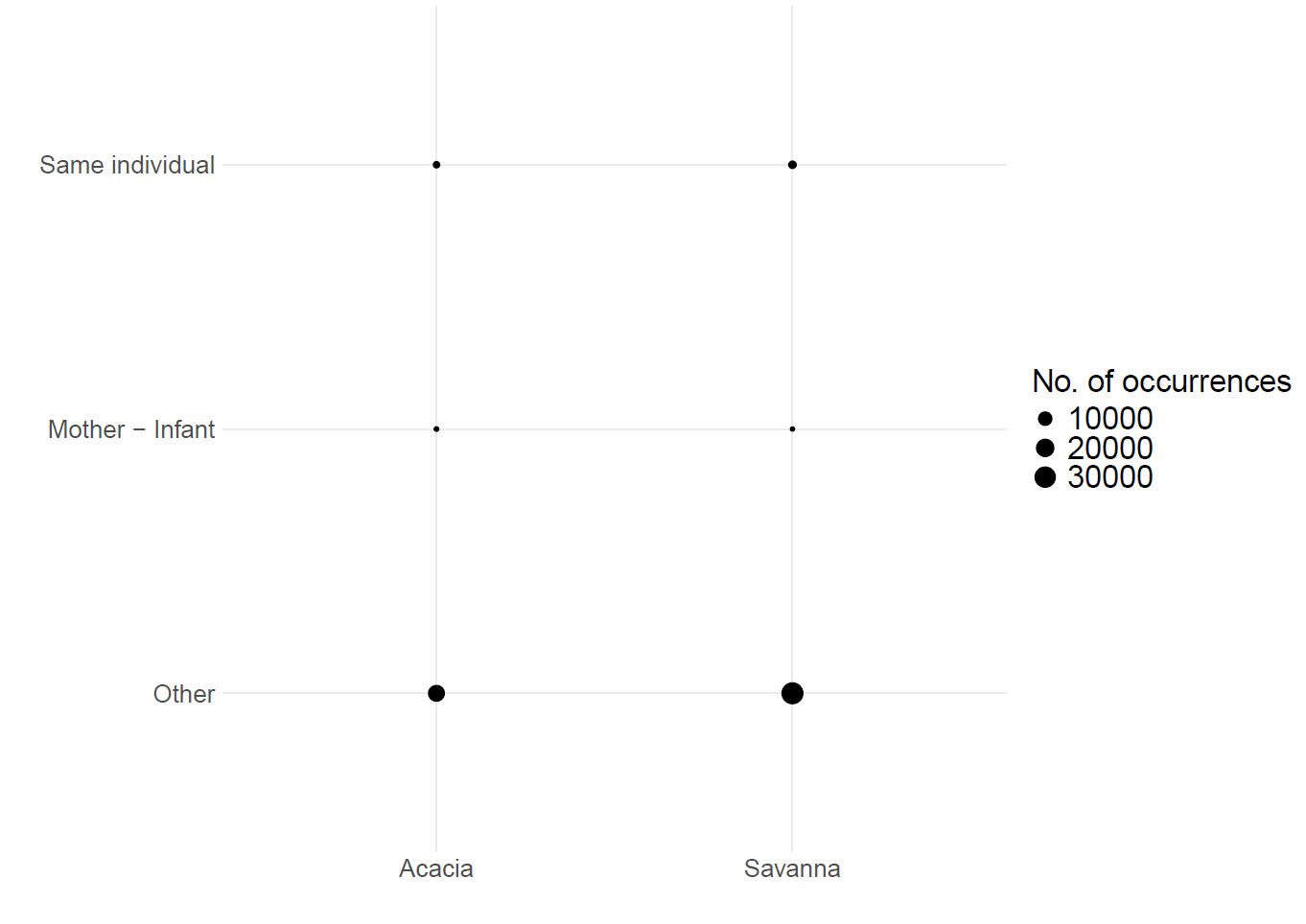


**Figure S3:** Number of occurrences for each relationship between faecal samples for Acacia on the left and Savanna on the right.


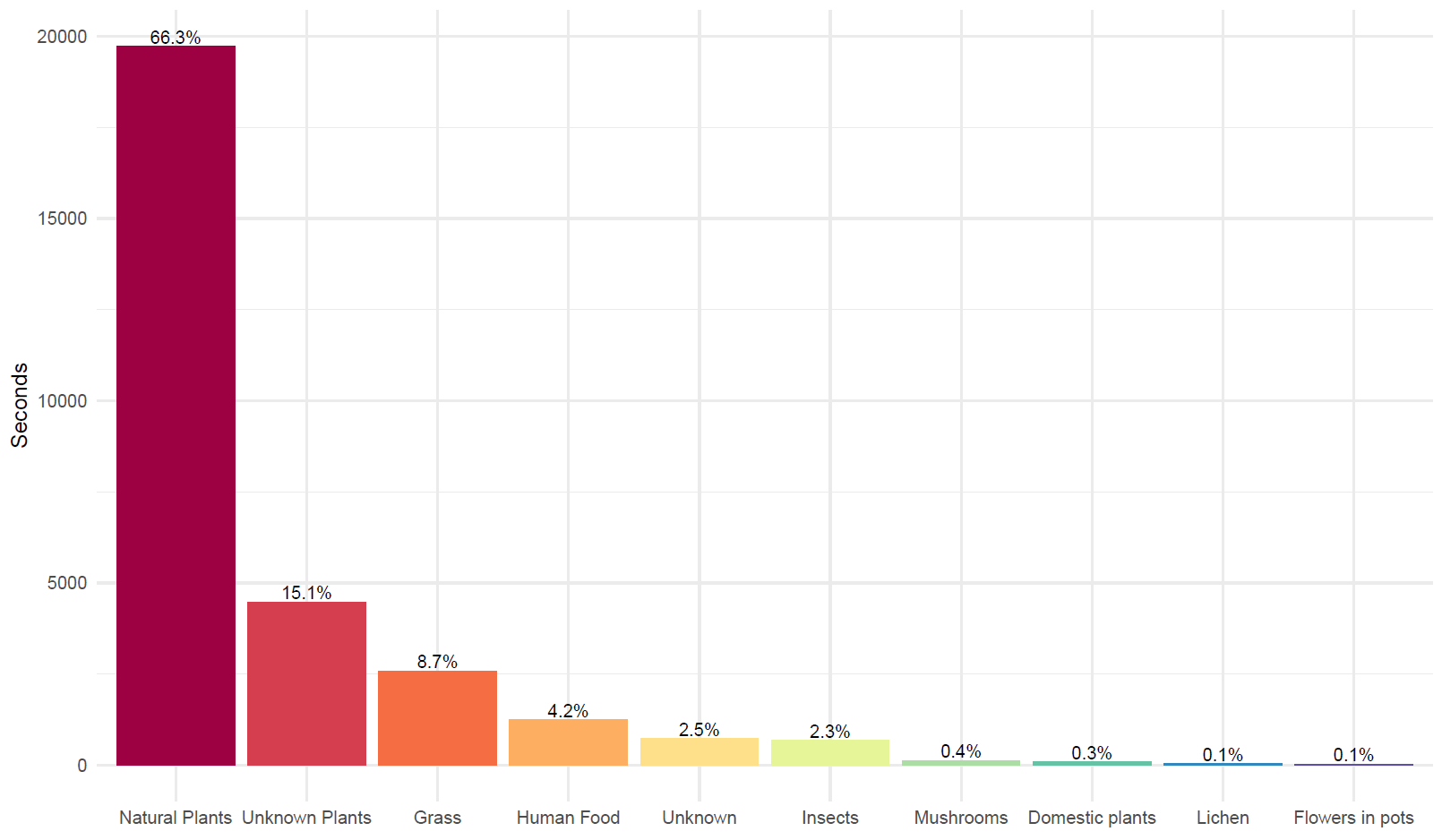
**Figure S4:** Foraging time per category for the two combined monkey groups based on observational data.


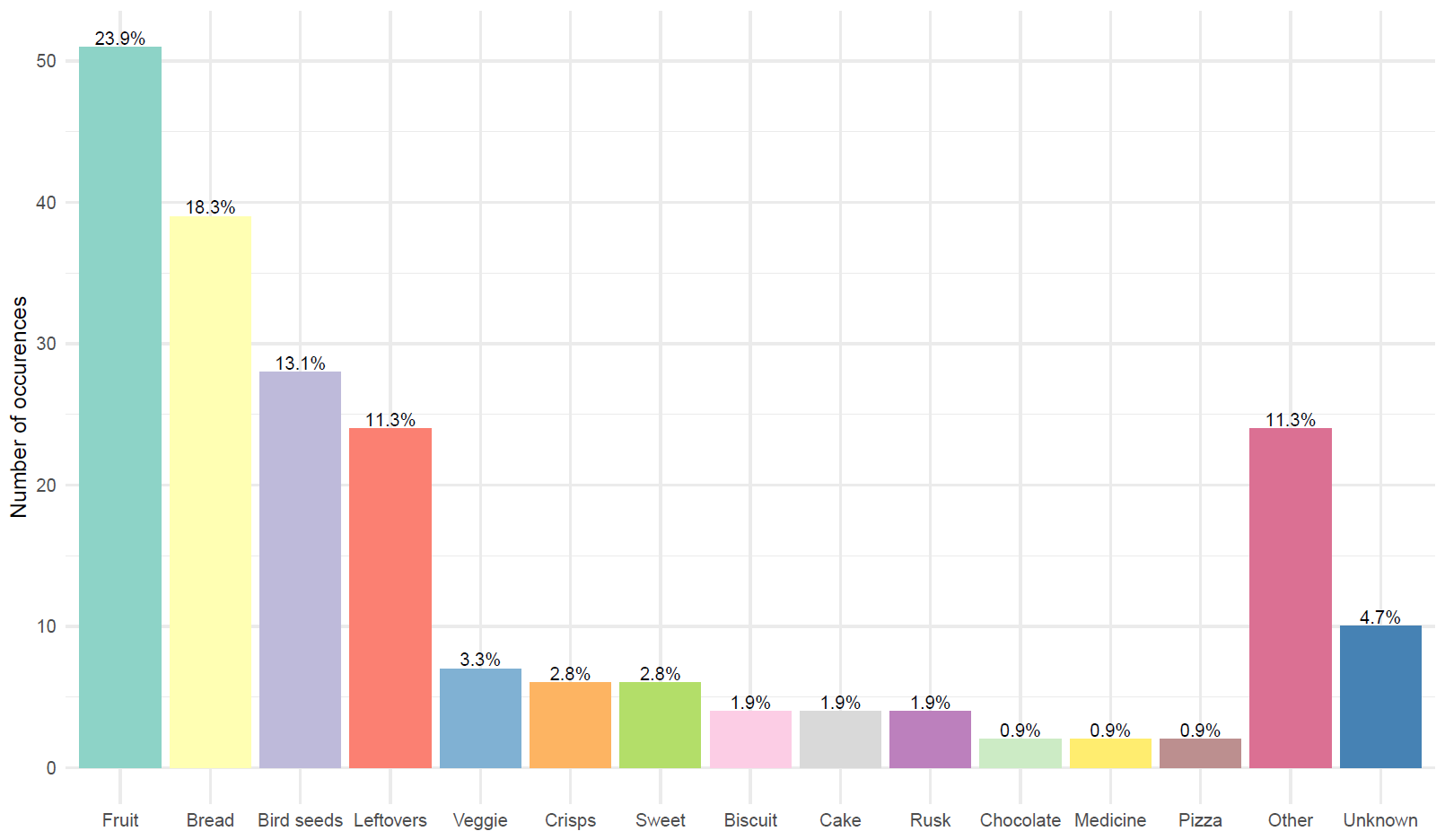


**Figure S5:** Number of occurrences of each anthropogenic food category for the two monkey groups combined based on ad libitum data.
